# Molecular Covalent Functionalization of Graphene and Its Derivatives: An Effective Strategy to Boost Electrocatalytic HER

**DOI:** 10.1021/acsomega.5c06793

**Published:** 2025-10-09

**Authors:** Shivani, Xuan Thang Cao, Pavel Kopel, Subodh Kumar

**Affiliations:** † Department of Inorganic Chemistry, Faculty of Science, 98735 Palacký University Olomouc, 17 Listopadu 12, 77146 Olomouc, Czech Republic; ‡ Faculty of Chemical Engineering, Industrial University of Ho Chi Minh City, Ho Chi Minh City 700000, Vietnam

## Abstract

Graphene-based electrocatalysts have been developed, and they exhibited enhanced activity due to their superior electronic conductivity. The robustness of such graphene materials can be further enhanced by altering their chemical and physical properties using different techniques. Molecular covalent functionalization is one of the effective strategies to alter the chemical composition, electronic structure, surface area, as well as dispersibility of graphene materials. Despite the significant literature on its contribution to improving the electrocatalytic activity for the hydrogen evolution reaction (HER), there is no review article available. Therefore, we have tried to fill this void by examining recent developments in the field of molecular covalent functionalized graphene and its derivatives for water electrolysis. We have also thoroughly discussed the role of individual components (graphene support, linker, and functional molecules bearing the main active sites) to improve the performance of the electrocatalyst by inducing synergistic effects and enriching surface properties. Moreover, the main characteristics of effective electrocatalysts, such as the surface area, functionality, dispersibility, conductivity, stability, and electronic structure, have also been reviewed. Finally, challenges and future perspectives are outlined to assist researchers in designing more effective electrocatalysts for the HER.

## Graphical abstract

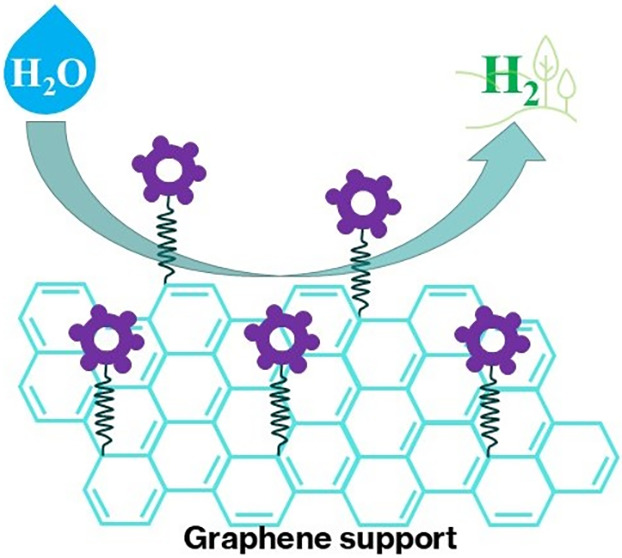

## Introduction

Humans have always needed substantial energy for continuous progress. However, the sources of energy are mainly based on fossil fuels and consequently have started to cause environmental problems due to the excess generation of greenhouse gases. Moreover, they will vanish someday as their abundance is limited; however, the demand for energy is increasing day by day. Therefore, alternative sources need to be extensively explored to produce enough clean energy to meet the needs of society. However, the production of clean energy is only logical if its sources are sustainable and produce no harmful byproducts. In this context, H_2_ is a potential candidate that produces only water on combustion, generating significant energy.^1^ It is naturally abundant and is being used as one of the high-energy sources.^2^ Yet again, its natural resources are also limited, and, therefore, researchers have started discovering other potential methods to produce H_2_.^3^ One of the promising strategies is water electrolysis, which is a beneficial approach in terms of the vast availability of starting reactants, i.e., water, and produces only O_2_ as a side product.^4–6^ During electrocatalytic water reduction, water molecules are reduced at the cathode and oxidized at the anode to liberate H_2_ and O_2_ at the respective electrodes. These reactions are called half-cell reactions: the hydrogen evolution reaction (HER) and the oxygen evolution reaction (OER). The kinetics of water splitting are sluggish due to the high overpotential. Therefore, the development of efficient electrocatalysts with superior conductivity, long-term stability, capability of withstanding high current density, and, of course, low cost is required.^7^ Generally, platinum (Pt)-based electrocatalysts are superior in terms of optimal hydrogen adsorption energy and current density for the HER in both media (acidic and basic).^8,9^ However, involvement of high-priced Pt in such electrocatalysts has made them a less favorable choice for commercial purposes. Therefore, nonnoble metal-based electrocatalysts^10^ (metal oxides,^11,12^ metal organic frameworks,^13^ nitrides,^14^ graphitic materials,^15,16^ phosphides,^17,18^ sulfides,^18,19^ and hybrid nanomaterials^20^) have been investigated and have shown comparative performance. However, lower selectivity and the instability of active sites in terms of corrosion, aggregation, leaching, and stacking of nanosheets are major concerns for long-term applications. The inherent characteristics of the materials used for the construction of electrocatalysts are some of the crucial factors for their appropriateness to produce H_2_ via water electrolysis.^21^ Graphitic carbon materials and their hybrids are well-known electrocatalysts for the HER due to their tunable nature in terms of conductivity and physicochemical properties.^22,23^ Moreover, their biodegradable nature, abundance, and low cost are added advantages. Graphene and its derivatives are two-dimensional (2D) graphitic materials with attractive properties (high conductivity, large surface area, superior mechanical strength).^24,25^ Derivatives of graphene include graphene oxide (GO),^26^ reduced graphene oxide (rGO),^27^ graphene quantum dots (GQDs),^28^ functionalized graphene, and doped graphene^29,30^ and are equally fascinating in the view of their structural merits. If we consider the research trend over the last 10 years, graphene-based electrocatalysts for the HER have been successively amplified, as indicated by the number of articles published each year (Figure 1). The publication of a large number of research articles indicates the great significance of graphene materials in developing HER electrocatalysts. Since the discovery of graphene, much research has been carried out in different areas. However, electrocatalysts and electronic areas have benefited the most from the unprecedented conductivity and tunable electronic properties of graphene. In the past decade, significant improvements have been made in the development of electrocatalysts in terms of stability and activity. Deng et al. have developed a hierarchical structure encapsulating a CoNi nanoalloy with ultrathin graphene layers to improve the HER performance, which was quite close to that of the commercial 40% Pt/C catalysts. The 1–3-layered graphene shells allowed electron movement from CoNi to the graphene surface, resulting in superior performance.^31^ Yue et al. have synthesized metal-free N- (pyridinic N-rich) and F-codoped porous graphene nanosheets. The catalytic performance of N active sites for the HER was promoted through F-codoping by lowering the Gibbs free energy.^32^ The involvement of single-atom catalysts in the development of electrocatalysts is imminent. Hossain et al. have designed a series of transition metal-based single-atom catalysts (SACs) deposited on N-doped graphene as SACs for the electrocatalytic HER to provide insight in developing highly performant SACs for the HER. Only Co, Fe, Cr, V, and Rh exhibited good performances. Co-SAC showed the highest electrochemical activity.^33^ Moreover, Su et al. have precisely modulated the electronic structure of O-doped graphene-supported metal nanoparticles (Ru) by incorporating different single metal atoms (Fe, Co, and Ni). The synthesized hybrid electrocatalysts exhibited superior HER performance compared to many previously reported ones, including state-of-the-art Pt/C.^34^ Vertical graphene sheets can offer a comparatively high surface area and fast electron transfer. Moreover, their multibranched nature can reduce the possibility of agglomeration. Wu et al. grew vertical graphene sheets using CVD and constructed a nanocomposite consisting of CoP nanoparticles deposited on N-doped vertical graphene sheets/carbon black. The assembled nanocomposites performed comparably to expensive Pt/C for the HER.^35^ In the current scenario, to achieve high stability without compromising HER activity for seawater is a challenge. Lin et al. have designed Mo_2_C–Ru catalysts with dual active sites by incorporating Ru and β-Mo_2_C nanoparticles. Graphene is then integrated for stability as well as facile electron transfer through interface engineering techniques. The resulting electrocatalyst impressively worked under alkaline seawater and outperformed commercial Pt–C.^36^ Generally, the 2D surface is easy to functionalize, and the resulting electrocatalyst can have more exposed and accessible active sites to the reaction substrates. Bulk synthesis of graphene derivatives is also possible, which is important for industrial applications. In fact, GO is commercially available and can be utilized as a starting material for the preparation of other desirable derivatives. There are various methods to develop graphene-based electrocatalysts, such as doping,^37^ metal nanoparticle deposition,^38,39^ molecular covalent functionalization,^40,41^ and physical attachment of catalytic molecules.^42^ Molecular covalent functionalization is one of the effective strategies to adjust the chemical composition, electronic structure, surface area, dispersibility, and morphology of graphene materials.^43,44^ It can determine the catalytic nature of the electrocatalysts.^45^ The synthesis of molecular covalently functionalized electrocatalysts includes the attachment of molecular entities (molecular complex,^46^ organic molecules,^47^ organic polymers,^48^ covalent organic frameworks,^49^ and metal nanoparticles^50^) onto the surface of graphene through covalent linkage. Nevertheless, additional entities, including other nanomaterials, can be attached to construct heterostructures involving more than one nanomaterial. This modification can integrate the virtues of each component and create enriched active sites that can participate in further enhancing HER activity. There are various reviews available discussing strategies to modify the graphene-based nanomaterials.^43,44^ However, to the best of our knowledge, no single review is primarily dedicated to conferring the significance of the molecular covalent functionalization approach for the preparation of high-performance electrocatalysts specifically for the HER.

**Figure 1:**
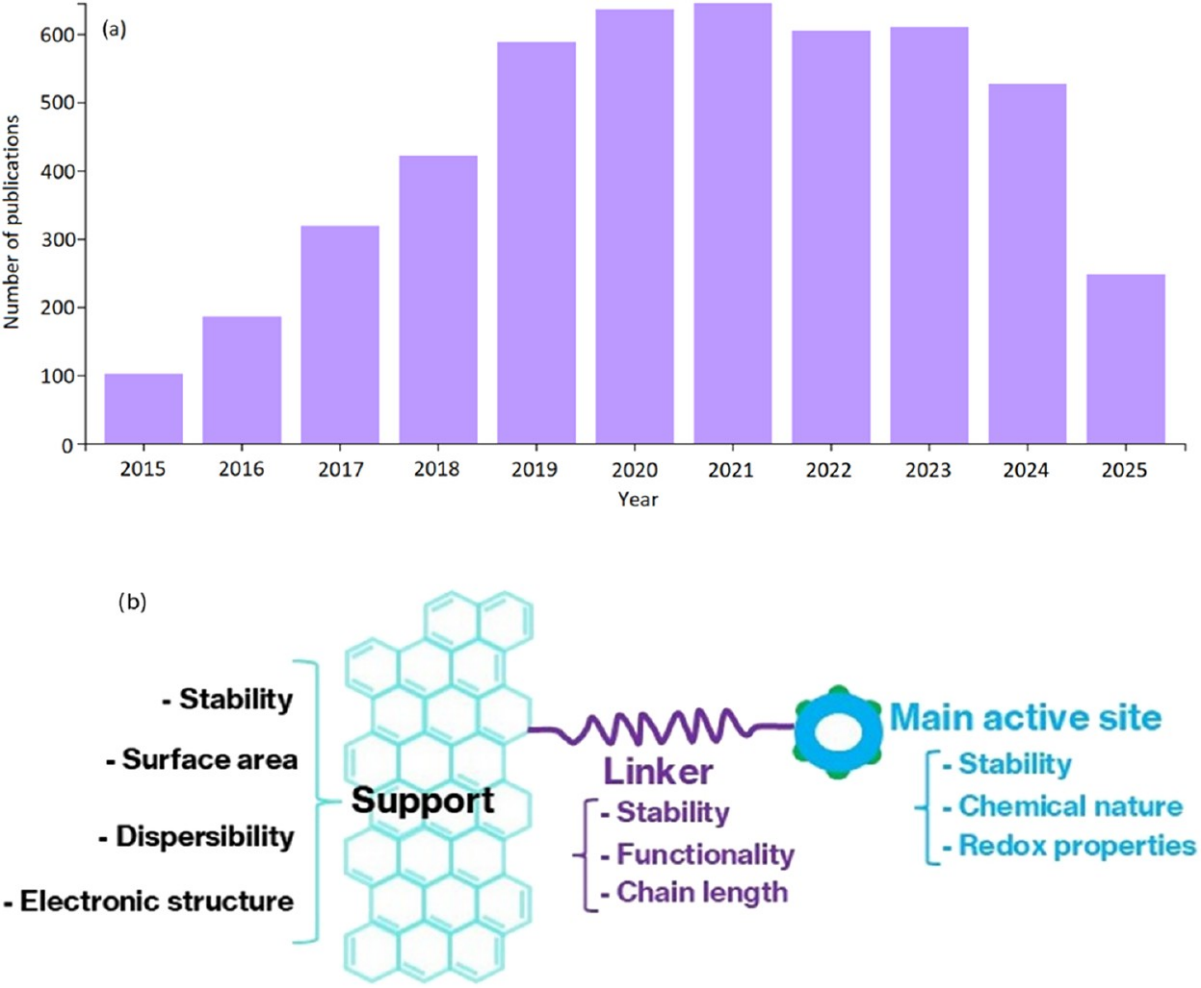
(a) Research trend over the last 10 years in terms of the number of publications published (source: Web of Science), and (b) graphene-functionalized active sites using an organic linker and their individual characteristics affecting catalytic activity.

In this review article, molecular covalently functionalized graphene-based electrocatalysts are thoroughly discussed, focusing on recent achievements in their synthesis and modification to improve their electrocatalytic activity for the HER. Researchers would certainly benefit from the centric approach of this review article while knowing the prospects and accompanying challenges in designing robust future electrocatalysts.

## Insights into Molecular Covalent Functionalization

Graphene nanosheets and their derivatives can be functionalized via covalent and noncovalent approaches. Covalent functionalization can improve the dispersibility and processability and introduce defects by establishing strong and stable chemical bonds with the carbon atoms of graphene. This method can significantly alter the electronic properties precisely as well as the graphene structure due to the controlled transformation of C sp^2^ to C sp^3^. Moreover, the covalent attachment of functional molecules provides better stability under challenging reaction conditions. On the other hand, noncovalent functionalization of graphene involves weaker interactions (π–π stacking, hydrogen bonding, electrostatic interactions, and van der Waals forces) to attach molecules to its surface. In this method, the intrinsic properties of graphene are preserved, but other properties, such as dispersibility, drug delivery, biosensing, and biocompatibility, are improved. However, it is generally not as stable as covalent functionalization. In fact, both functionalization methods are used, depending on the target application. Molecular covalent functionalization is one of the effective and systematic strategies to exploit the potential of support materials as well as attached catalytic entities (functional molecules, metal ions, and other nanomaterials).^51–53^ This whole process involves the deliberate addition of catalytic entities to the surface of support materials with or without involving linker molecules. However, linkers are not necessarily always a part of the synthesis of molecular covalently functionalized electrocatalysts. Functional molecules can themselves be directly attached via various chemical reactions.^43^ This methodology is targeted to improve stability and electrocatalytic activity by adjusting not only the electron transfer properties but also the spatial arrangement of active sites. It can amend the chemical, electronic, and surface properties of the resulting hybrid materials by integrating the individual characteristics of the participants (support material, linkers, and functional molecules/nanomaterials).^54^ Hence, the molecular covalently functionalized catalysts can exhibit synergistic effects due to the establishment of a novel interface between the attached catalytic entities and substrate, thus resulting in superior performance compared to separate individuals. Moreover, it benefits from generating well-organized active sites to maximize their exposure to the reactants and intermediates. The significance of this study further includes the reduction in the leaching of active sites and their stacking nature, leading to a long-lasting performance with higher efficiency. There are different ways, such as chemical,^55,56^ hydrothermal,^57,58^ plasma-assisted,^59^ and ball milling,^60,61^ to perform the molecular covalent functionalization on the surface of graphene-based materials.^62,63^ These methods can be selected based on the nature of support materials, the requirement of specific functional groups, and targeted applications. However, some conventional processes suffer from the complexity of the reactions involved, use of harmful chemicals, cost, and challenging scale-up. Although all of the methods are intended to generate different kinds of molecular functionalities on the graphene surface through covalent bond formation, chemical methods are generally applied. For instance, graphene-based materials can be modified by applying different chemical approaches (e.g., amination,^64,65^ silanization,^66,67^ diazotization,^68^ cyanation,^69^ sulfonation,^70^ and cycloaddition^71^)^52^ depending on the availability of native functionality on their surface.^63^ Moreover, each chemical approach has its own significance and associated drawbacks. For example, GO, the oxidized form of graphene layers, has ample oxygenated functional groups (carbonyl, carboxyl, hydroxy, and epoxy) on the basal plane and at the edges and can directly participate to form a covalent linkage with guest molecules bearing suitable functional groups. On the other hand, graphene and reduced graphene oxide (rGO) have very limited or no oxygen functionalities and less dispersibility in various solvents; consequently, they cannot effectively contribute to forming covalent bonds. Therefore, such graphene nanomaterials need to be prefunctionalized via oxidation, diazonium, and cycloaddition reactions to generate chemically suitable functionality on their surface by forming mainly C–O, C–N, and C–C bonds (Figure 1).^43,44^ Such intentional modifications can be used as active sites themselves and as anchoring points for the further functionalization of desirable molecules. Oxidative functional groups are comparatively easy to grow on graphene supports by performing harsh oxidation reactions using inorganic acids and strong oxidants.

These oxygenated groups can easily react with other linkers, such as silanes, to provide more accessible functional sites by forming desirable covalent linkages. The diazonium reaction is one of the important alternatives to attach an aromatic amine molecule bearing other functional groups directly onto the graphene surface. In this method, the amine group is primarily converted into diazonium salt in the presence of sodium nitrate and acid, which in turn reacts with the graphene surface, forming an aryl radical and liberating nitrogen gas. The *in situ* formed aryl radical then reacts with the carbon of graphene, constructing a C–N bond. Hence, we can introduce an extensive range of functionalities to the graphene surface just by changing the functionality of the aryl amine and silane molecules. The Diels–Alder reaction is also an emerging reaction in the field of nanomaterial functionalization to attach organic molecules to the graphite surfaces. In this reaction, the C=C double bond reacts with appropriate molecules bearing diene functional groups, forming covalent bonds to give functional materials. Recently, cyanation of the graphene surface has been achieved by the substitution reaction using fluorinated graphene as a support material.^69^ CN groups were generated from the NaCN, which subsequently substituted the F-atoms from the graphene surface through a nucleophilic substitution reaction. However, these chemical reactions generally involve harmful chemicals and are not desirable in terms of environmental and human safety. Therefore, it is always advantageous to follow the path of less harmful but effective chemical routes. For example, the Diels–Alder reaction can also be conducted in deep eutectic solvents, excluding the harmful chemicals and inducing the desirable functionality in the resulting materials.^72^ Chemical methods are more suitable for the successive covalent functionalization of nanomaterials. On the other hand, hydrothermal, ball milling, and plasma-assisted methods are less common and are generally used to create molecular functionality in a single step. Graphene-based materials have been decorated via different routes by attaching various guest moieties (organic molecules, molecular complexes, covalent organic frameworks, and other nanomaterials), aiming to enhance the overall electrocatalytic activity for the HER (Table 1). Importantly, molecular covalent functionalization can provide a controlled and uniform distribution of the active sites on the graphene surface. Hence, there is a huge scope for modifying the surface of graphitic nanomaterials by precuring various graphene supports, organic linkers, and catalytic molecules (active sites) to make them holistic solutions for the plaguing applications.

**Table 1:** Graphene Oxide and Derivatives Based Covalent Functionalized Electrocatalysts for the HER

sn	catalysts components	method	HER activity	refs
1.	-GO	chemical	overpotential: 376 mV	^55^
	-terephthaloyl chloride		Tafel value: 241 mV dec^–1^	
	-porphyrin		RE: Ag/AgCl	
			CE: Pt	
			WE: GCE	
			medium: acidic	
2.	-GO	chemical	onset potential: 496 mV	^56^
	*-p*-phthalaldehyde		overpotential: 383 mV	
	-terephthaloyl chloride		Tafel value: 231 mV dec^–1^	
	-ZnTAPc		RE: Ag/AgCl	
			CE: Pt	
			WE: GCE	
			medium: acidic	
3.	-GO	chemical	onset potential: 474 mV	^56^
	-*p-*phthalaldehyde		over potential: 404 mV	
	-terephthaloyl chloride		Tafel value: 216 mV dec^–1^	
	-ZnTAPc		RE: Ag/AgCl	
			CE: Pt	
			WE: GCE	
			medium: acidic	
4.	-graphene aerogel	chemical	overpotential: 275 mV	^77^
	–1,3,5-triformylbenzene		Tafel value: 139 mV dec^–1^	
	–2,2′-bipyridine-5,5′-diamine		RE: Ag/AgCl	
	-aminopropyltriethoxysilane		CE: carbon rod	
			WE: GCE	
			medium: alkaline	
5.	-graphene quantum dots	chemical	overpotential: 433 mV	^135^
	-*N*-hydroxysuccinimide		Tafel value: 65 mV dec^–1^	
	-ethylenediamine		RE: Ag/AgCl	
			CE: Pt wire	
			WE: GCE	
			medium: alkaline	
6.	-HOPG	electrochemical	onset potential: 250 mV	^134^
	-porphyrin		overpotential: 530 mV	
			Tafel value: 128 mV dec^–1^	
			RE: Ag/AgCl	
			CE: Pt wire	
			WE: GCE	
			medium: acidic	
7.	-GO	chemical, electrochemical	onset potential: −0.7 V	^82^
	-cobalt bis(benzylammoniumdithiolate)		TOF: 105 s^–1^	
			Tafel value: 59 mV dec^–1^	
			RE: Ag/AgCl	
			CE: carbon rod	
			WE: GCE	
			medium: acidic	
8.	-GO	chemical	overpotential: 47 mV	^136^
	-oleyl amine		Tafel value: 64 mV dec^–1^	
	-CZTS (Cu_2_ZnSnS_4_)		RE: SCE	
			CE: Pt wire	
			WE: GCE	
			medium: acidic	
9.	-GO	chemical, electrochemical	overpotential: 161 mV	^137^
	-PdCl_2_		Onset potential: −0.205 V	
	–2-pyridinecarboxaldehyde		Tafel value: 138 mV dec^–1^	
	–3-aminopropyltriethoxysilane		RE: Ag/AgCl	
			CE: Pt wire	
			WE: GCE	
			medium: acidic	
10.	-GO	chemical, electrochemical	overpotential: 148 mV	^137^
	-PdCl_2_		onset potential: −0.190 V	
	–2-pyridinecarboxaldehyde		Tafel value: 129 mV dec^–1^	
	–3-aminopropyltriethoxysilane		RE: Ag/AgCl	
			CE: Pt wire	
			WE: GCE	
			medium: acidic	
11.	-GO	hydrothermal	overpotential: 39.6 mV	^57^
	-PdCl_2_		Tafel value: 29.7 mV dec^–1^	
	-poly(*N*-vinylpyrrolidone)		RE: Ag/AgCl	
			CE: Pt wire	
			WE: GCE	
			medium: alkaline	
12.	-GO	chemical	overpotential: 254 mV	^70^
	-sulfur powder		onset potential: 97 mV	
	-sodium sulfide nonahydrate		Tafel value: 81 mV dec^–1^	
			RE: Ag/AgCl	
			CE: Pt	
			WE: GCE	
			medium: acidic	
13.	-pristine GNP	plasma	overpotential: 220 mV	^59^
			Tafel value: 117 mV dec^–1^	
			RE: Hg/Hg_2_SO_4_	
			CE: graphite rod	
			WE: GCE	
			medium: acidic	
14.	-GO	hydrothermal, ball milling	overpotential: 350 mV	^60^
	-KOH		onset potential: 100 mV	
	-ammonia		Tafel value: 113 mV dec^–1^	
			RE: SCE	
			CE: carbon rod	
			WE: GCE	
			medium: acidic	
15.	-GO	hydrothermal	overpotential: 200 mV	^58^
	-(NH_4_)_2_MoS_4_		onset potential: 90 mV	
	-O-MWCNT		Tafel value: 48 mV dec^–1^	
	-*p-*phenylenediamine		RE: SCE	
			CE: Pt slice	
			WE: GCE	
			medium: acidic	
16.	-NRGO	chemical	overpotential: 506 mV	^138^
	-graphitic carbon nitride		onset potential: 0.360 V	
	–1-hydroxybenzotriazole		Tafel value: 147 mV dec^–1^	
	-*N*,*N*-diisopropylethylamine		RE: Ag/AgCl	
			CE: Pt coil	
			WE: GCE	
			medium: acidic	
17.	-GO	chemical	onset potential: −50 mV	^103^
	-polyDADMAC		Tafel value: 65 mV dec^–1^	
	-exfoliated MoS_2_		RE: Ag/AgCl	
			CE: Pt coil	
			WE: GCE	
			medium: acidic	
18.	-GQD	hydrothermal	overpotential: 245 mV	^139^
	–1,3,6-trinitropyridine		Tafel value: 93.2 mV dec^–1^	
	-thiourea		RE: Hg/Hg_2_Cl_2_	
	-Na_2_SO_3_		CE: graphite rod	
			WE: GCE	
			medium: acidic	
19.	-GQD	mechanochemical	overpotential: 270 mV	^61^
	-MoS_2_		Tafel value: 83.3 mV dec^–1^	
			RE: Hg/Hg_2_Cl_2_	
			CE: graphite rod	
			WE: GCE	
			medium: acidic	
20.	-GQDs	chemical, hydrothermal	overpotential: 189 mV	^28^
	-CoP		Tafel value: 93 mV dec^–1^	
	-TiO_2_		RE: SCE	
			CE: graphite plate	
			WE: GCE	
			medium: basic	

## Impact of Components on the Electrocatalytic Activity

Molecular covalent functionalization requires mainly three components such as graphene supports, organic linkers, and molecules bearing active sites (functional molecules) (Figure 2). All these individual components can also contribute to enhancing the electrocatalytic activity of the resulting materials. Although functional molecules may directly be attached covalently to the surface of the support, it is not always possible due to the lack of desired functionality and, thus, may require a linker. Moreover, linkers can also be used to enhance the number of adsorption sites. The inherent properties of these individual components are integrated, which may cause a synergistic effect and astonishingly accomplish superior activity. Their selection is made based on the requirements of the target application. In general, functional polymers are used to enhance the number of adsorption sites and porosity of the materials.^30^ Homogeneous metal complexes are anchored to make them heterogeneous in nature.^73^ Other metal ions are also bound to the functional sites on the support materials to construct an inexpensive heterogeneous active center.^74,75^ Other nanomaterials such as metal organic framework (MOF),^76^ covalent organic framework (COF),^77^ carbon nitride,^78,79^ and 2D metal sulfides^80,81^ are also covalently immobilized onto the other nanomaterials, including graphene derivatives, to enhance the electron transfer to facilitate the HER. Ultimately, the nature of the functional molecules and established covalent bonds can adjust the electronic properties of the active sites to improve the overall activity and selectivity of the electrocatalyst. Although covalently immobilized molecules bearing active sites are the main entities, they are not solely responsible for the catalytic activity.^82^ In this section, we will discuss the properties and roles of all individual participants responsible for improving the HER activity.

**Figure 2:**
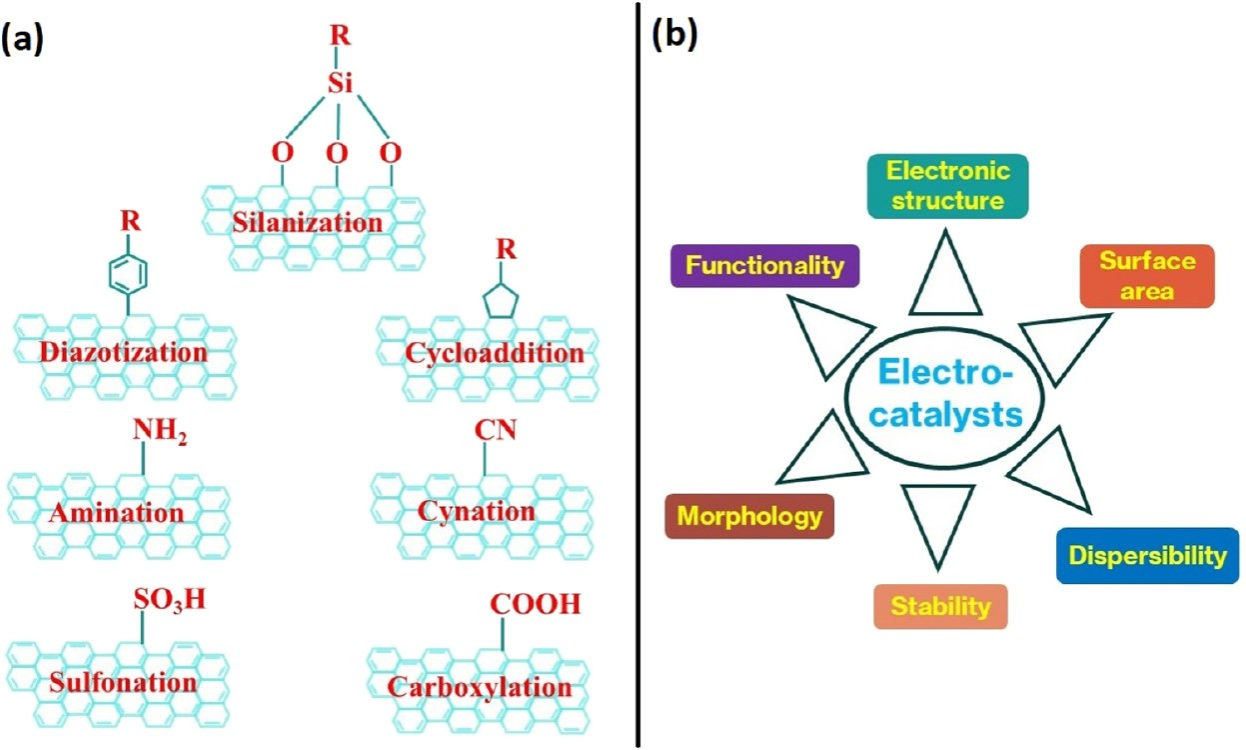
(a) General methods for the molecular covalent functionalization of graphene. (b) Properties of efficient electrocatalysts for the HER.

### Graphene Support

Graphene, rGO, GO, GQDs, and fluorographene (FGr) are 2D carbon materials with distinct properties in terms of conductivity, dispersibility, functionality, stability, and surface area, which are the primary conditions for an effective electrocatalyst (Figure 3).^83–86^ All of these materials are used to prepare diverse types of electrocatalysts. Graphene is generally synthesized by top-down approaches, by the physical and chemical exfoliation process, and a bottom-up approach, such as chemical vapor deposition (CVD).^87^ Although the top-down approach can produce comparatively higher amounts of graphene than the CVD method, the quality of graphene is poor in terms of thickness (high number of graphene layers), defects, and electronic nature. CVD can produce single-layer to few-layer graphene, but its quantitative production is a limitation for some commercial electrocatalysis applications. Therefore, rGO is more favorable as it has similar properties to graphene and can be synthesized in bulk by the reduction of GO via various approaches.^88^ GQDs are produced by the chemical and physical scissoring of graphene oxide nanosheets and can have distinct electronic structures due to the quantum effects.^89^ Interestingly, these tiny, conjugated carbon nanosheets can also be further functionalized. Hence, the chemical functionality and size effects can affect the final catalytic activity of the material. Fluorographene is also one of the most demanding supports and can be produced by the exfoliation of fluorinated graphite. F-atoms on the surface of fluorographene can be easily substituted by different groups, such as amines and CN, under moderate reaction conditions, yielding desirable functionalized graphene nanosheets.^90,91^ However, GO remains the most desirable precursor to synthesize various kinds of electrocatalysts through covalent functionalization due to its bulk synthesis, native oxygenated functional groups (−COOH, −OH, and epoxide), application of the reductive form (rGO), and easof functionalization.^92^ Importantly, GO synthesized by different methods may have different degrees of defects, dispersibility, nanosheet size and thickness, functionality, and electronic structure.^93^ Accordingly, the synthesis process of the graphene support can not only significantly affect their covalent functionalization process but also participate in determining the reaction mechanism of the electrochemical reaction.^94^ Therefore, it is necessary to verify the native characteristic properties of GO before utilizing them as support materials, as different GO materials can result in different electrocatalytic activities, even for similar covalent functionalization processes and attached catalytic molecules.^95,96^

**Figure 3:**
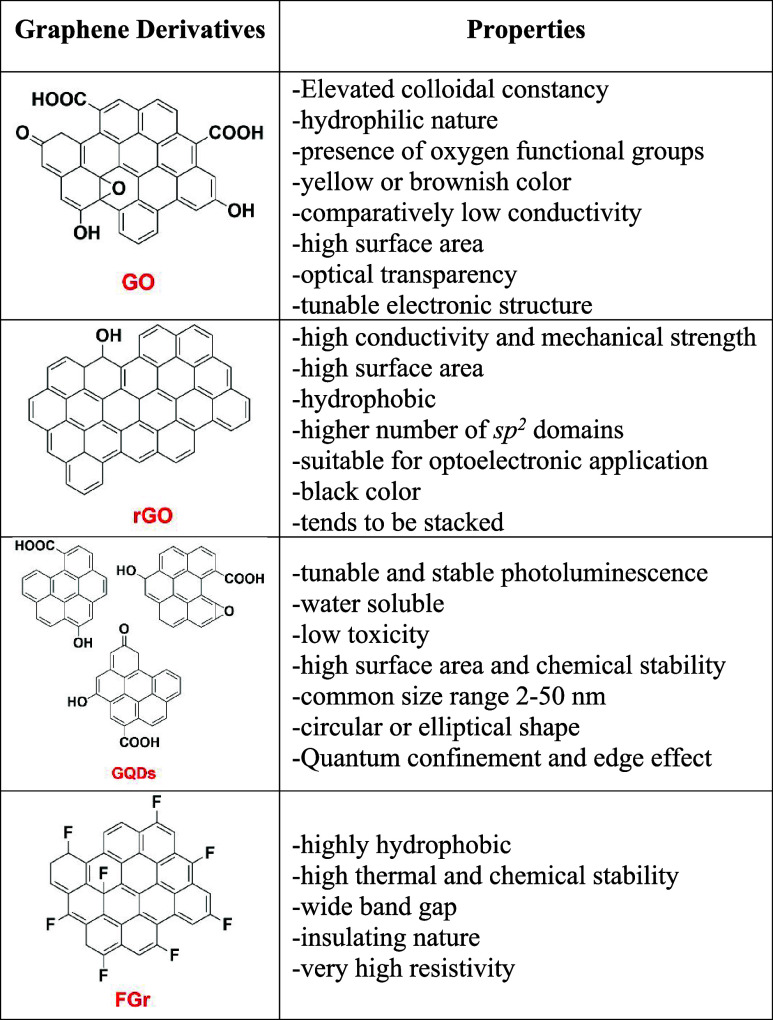
Structure and properties of graphene oxide (GO), reduced graphene oxide (rGO), graphene oxide quantum dots (GQDs), and fluorographene (FGr).

### Organic Linkers

Organic linkers are the bifunctional or multifunctional organic molecules that are used to prefunctionalize the graphene surface or their derivatives to generate or increase the number of desirable binding sites for further functionalization.^97^ There are different types of organic linkers. In fact, any bifunctional organic molecule bearing desirable functionality at a suitable position can be used as a linker. In most cases, some known linkers include silanes,^98^ amines,^99^ and bifunctional organic molecules.^100^ Sometimes, one or more linkers are needed to make a binding site, such as a Schiff base. Schiff bases are one of the most used functional groups for the complexation of a wide range of metal ions.^101^ However, selecting an appropriate linker can be complicated as it should be inexpensive and stable in nature, reactive toward functional groups of binding entities, and carry desirable functionality. They are needed to achieve effective and stable covalent functionalization for the desired application and can be directly involved in the electron transfer process, besides merely holding the molecules bearing active sites. The linker acts as a bridge between the substrate material and active sites while being stable under the reaction conditions. Importantly, their chemical nature can also modify the electronic properties of the support materials and active sites. The electron-donating and electron-withdrawing molecules have shown different performances in the electrocatalytic reactions.^102^ In fact, electron-donating molecules are known to enhance electron conductivity.^60^ Moreover, we can also generate negative or positive charges on the support materials by attaching differently charged organic molecules. These oppositely charged support materials can be used to fabricate various types of nanocomposites through electrostatic attraction and have their own significance to integrate the properties of participating nanomaterials.^103^ Moreover, the availability of residual functionality on linker molecules may act as an adsorption site and facilitate electron transportation. Linker molecules can have different degrees of reactivity and innerness toward the functional groups of different substrates to form covalent linkages. Therefore, it is also important to assess the stability of the formed linkage in terms of the possibility of reactions between the reaction substrates and the linkage functional group. This might cause the breaking of the covalent linkage, resulting in the degradation of the catalysts, thus making them unsuitable for long-term reactions. The resulting covalent linkage is dependent on the functionality of the linkers and support and can be predicted while designing the electrocatalysts. Mostly, amide, Schiff base, ether, and ester-type covalent linkages are formed as they remain stable during electrochemical reactions and sometimes participate in enhancing the reaction rate. Besides the chemical functionality, the length of the linker chains is also crucial and can affect the electron transfer process as well as stability. Generally, longer chains are not suitable and can restrict the exposure of active sites due to steric hindrance and delay electron transfer, leading to comparatively lower reaction rates. Thus, an ideal chain length is required to exploit a higher number of active sites without any stacking and electronic resistance to accelerate the electrochemical reaction.^104^ Accordingly, we can tune the electrocatalytic activity of the electrocatalysts by adjusting the lengths of the linkers.

### Catalytic Molecules (Active Sites)

Active sites, where electrochemical reactions occur, can be metallic or nonmetallic and are inherited in the form of functional groups and metal centers in different electrocatalysts. The systematic modulation of the redox potential and electronic density to improve the overall electronic properties of the active sites is crucial for influencing electrochemical reaction pathways. Moreover, the presence of two or more active sites can participate synergistically to improve the performance of the resulting electrocatalysts. Generally, electrocatalysts are organic molecules,^105^ polymers,^106^ molecular complexes,^107^ metals and their oxides,^108^ and carbon nanomaterials and their heterostructures.^109^ Organic molecules consisting of N–, S–, O–, and P– can act as electrocatalysts and are well documented in the literature in the form of nitrogenated heterocyclic molecules.^110^ Their tunable structures, comparatively less expensive, and a metal-free nature distinguish them from other catalysts. Organic polymers suffer from lower degrees of conjugation and lower solubility. Low conjugation in the support material leads to poorer catalytic efficiency for the HER due to the hindrance of charge transfer and electronic interaction between the catalyst active sites and reaction intermediates required for the electrochemical steps. On the other hand, covalent organic frameworks (COFs) and metal organic frameworks (MOFs), types of ordered organic polymers, have good activity toward electrocatalytic applications.^111,112^ These materials can be further functionalized onto the graphene support to enhance the overall conductivity, selectivity, and stability of the electrocatalysts. Importantly, the selection of linkers and metal ions (in the case of MOF) can determine the electronic structure of the electrocatalyst. These immobilized organic frameworks can offer facile accessibility of active sites to the reaction substrates due to their high surface area and precisely tunable porosity. More importantly, such polymeric materials can be designed in terms of the structure, functionality, and pore size distribution based on the reaction substrates. Molecular complexes have proven to be efficient and selective electrocatalysts, offering precise generation of active sites with specific electronic structures. They can be designed with specific geometries to be more stable and selective in electrochemical reactions. Some molecular complexes, even those with metals of high abundance and lower cost, have shown comparable performance to metal complexes involving precious metals. However, their homogeneous nature restricts their recyclability, which not only increases the cost of the electrochemical process but also contaminates the products. Hence, covalent functionalization can deliver an effective way to utilize the full potential of metal complexes with superior activities on the surface of graphene.^43^ Moreover, it can provide precise control over their distribution while recycling them. Metal oxides are an alternative class of cost-effective electrocatalysts to the precious metal-based electrocatalysts.^113^ They can facilitate oxidation and reduction reactions due to their tunable properties. Their chemical composition and morphology can be specifically tuned to achieve the desired electronic structure, which in turn can improve the reaction rate and selectivity. Importantly, their stability is impressive, even at high potentials and harsh corrosive reaction conditions, which are necessary for long-term commercial applications. Moreover, metal oxides can be further modified through various approaches to obtain surface functionalization and interface engineering to establish synergistic interactions.^114^ Generally, materials with a tunable electronic nature are promising for electrocatalysis. Metal nanoparticles (MNPs) can exhibit a high surface area and a high surface area-to-volume ratio, along with a tunable electronic structure, thus enhancing the electrocatalytic performance. Importantly, the morphology of MNPs can be precisely tuned so as to provide the electronic and catalytic properties, allowing them to achieve improved performance. However, bare MNPs are prone to aggregate and thus can lose their activity. Therefore, covalent functionalization strategies can be applied to restrict their aggregation by deposition onto a prefunctionalized graphene surface.^115^ Moreover, prefunctionalization with desirable functional molecules can tailor the uniform distribution, stability, and morphology of MNPs, leading to better activity and selectivity. Heterostructures are suitable combinations of two or more distinct materials with diverse properties. These heterostructures offer superior performance compared to their individual counterparts due to the developed synergistic effects, which lead to improved electron transfer and selectivity.^116,117^ Moreover, the interface generated between the two components plays an important role in controlling the reaction substrate to enhance the kinetics of the reaction. The strategy of heterostructure formation is not only to enhance the activity and stability but also to reduce the total cost of the catalyst by including inexpensive materials, which might not perform better when used alone. Hence, all of these bare components bearing active sites can exhibit electrocatalytic activity. However, sometimes it is inadequate due to its lower stability, poor selectivity, and insufficient conductivity. Molecular covalent attachment methods can overcome these hurdles to achieve optimum efficacy by forming covalent linkages between these components.

## Characteristics of Efficient Electrocatalysts

Designing an efficient electrocatalyst requires maximizing the efficiency, stability, selectivity, and durability while producing at a comparatively lower cost. To design such electrocatalysts, factors such as conductivity, chemical nature, surface area, morphology, stability, and dispersibility should be accounted (Figure 4). These factors have been identified based on the available literature and significantly influence the electrocatalytic performance.^7,118^ In this section, we will discuss the aspects of all of these factors in detail.

**Figure 4:**
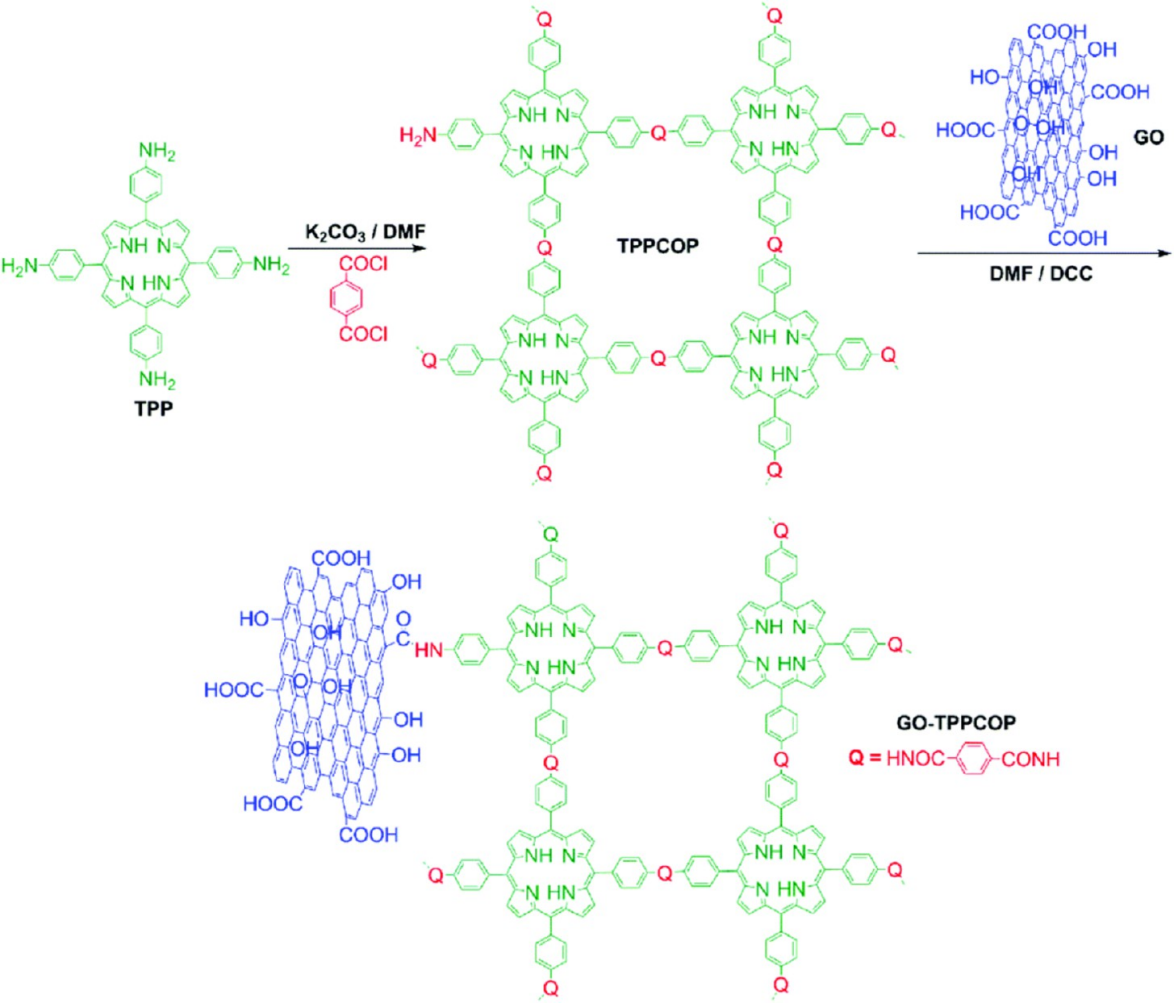
Synthetic routes for TPPCOP and GO-TPPCOP. Adapted with permission from ref 55. Copyright 2021, Royal Society of Chemistry.

### Morphology of Graphene Nanosheets

Graphene and its derivatives have a high exposed surface area and can be functionalized easily by the molecular covalent functionalization method, as already discussed in the previous section. As far as the morphology of graphene derivatives is concerned, the lateral size of the nanosheets and their stacking thickness are important in determining their activity. Moreover, the highly torn nanosheets can behave differently due to the availability of different degrees of edge and surface defects in the form of vacancies and voids that can induce porosity to the material.^119^ Importantly, the density and type of native defects in the graphene nanosheets are basically dependent on their synthesis process. In fact, in the case of graphene oxide, the degree of oxidation, size of sheets, and thickness are found to be dependent on the synthesis process.^96^ Subsequently, the sp^3^ to sp^2^ carbon ratio can also be affected. These native defects can also act along with active sites to enhance the activity by participating in the absorption/desorption and electron transfer processes. However, it is important to control the number of defects as they can also have adverse effects. Although it is difficult to precisely control their formation and density, tuning their type and abundance to target the electrochemical reaction would help construct an efficient electrocatalytic system. It is reported that the size of graphene nanosheets also affects their electrolytic properties.^120^ Graphene nanosheets with different lateral sizes may accommodate different degrees of defects and density of edges, thus enabling smooth interactions with reactant molecules. For instance, small nanosheets have a comparatively higher number of edges and a high surface area, which may help achieve superior dispersibility in different solvent systems. However, they can suffer from reduced stability and limited diffusion pathways, thus affecting their long-term performance. On the other hand, larger lateral-sized graphene nanosheets can carry larger pores, facilitate mass transfer, and provide an ample exposed surface for further functionalization with other molecules. However, the size of the graphene sheets needs to be optimal and mainly engineered to target electrochemical reactions for optimal performance.^62,121^ GQDs are the main example of size effects showing superior activity by direct participation in enhancing the catalytic activity. GQDs can be in the range from 2 to 20 nm and further covalently anchored on other materials to enrich the core characteristics of the subsequent materials.^89^ Hence, controlling the size and amount of defects can assist in achieving optimum activity. Moreover, bare graphene nanosheets are prone to stacking that can restrict access to active sites, hinder the diffusion of electrolytes, and reduce the surface area. Modulation of the interlayer distance between graphene sheets is desirable to modify the electrocatalytic nature and efficiency of electrochemical devices.^122^ Stacking is likely to occur due to the π–π interactions between the layers. Generally, mechanical and chemical exfoliation processes are found to be effective in adjusting the interlayer distance by placing a molecule between nanosheets, leading to the formation of exfoliated nanosheets. In this case, the size of the positioned molecules can determine the extent of interlayer spacing, which can be done via covalent or physical attachment. Both methods can manipulate the degree of stacking and have their own pros and cons. On the other hand, the removal of functional groups from the graphene surface can also adjust the interlayer distance between nanosheets. For instance, the controlled reduction of GO into rGO can also regulate its stacking nature. Hence, selecting the appropriate functional molecules in terms of size and stereo structure can not only reduce stacking but also modulate their interlayer distance.^123^

### Electronic Structure

A favorable electronic structure is a prime requirement for efficient electrocatalysis. The introduction of exotic functional molecules with fascinating features that are capable of altering the electronic properties of graphene derivatives is a prerequisite. In fact, electron-donating groups are fascinating to enhance the conjugation of the graphitic matrix by donating lone-pair electrons, which in turn improves the electric conductivity due to the strengthened density of states. In addition, electronic coupling is also a significant factor that cannot be ignored when discussing the improvement in the electronic properties of graphitic materials. The electronic structure can be used to determine the real potential of a material to execute an electrochemical reaction by interacting with the reaction substrates and enabling efficient electron transfer. Consequently, we can control the adsorption of reactants on the electrocatalyst surface by adjusting their electronic structure. However, an equilibrium between the adsorption of reactants and desorption of products is necessary for boosted performance and improved selectivity. Hence, electronic structure modulation is the key to directing the reaction kinetics by limiting the electron exchange between the electrode and reaction substrate. The electronic structure can be optimized by doping, interface engineering, forming intentional defects, and modulating active sites. These processes generally involve the introduction of impurities in the form of metal or nonmetal atoms and molecular functionality to the graphene lattice to generate new energy levels to facilitate electron transit more easily. It can influence the concentration of charge carriers and, consequently, the electrical conductivity based on the nature of functionalized entities (n-type or p-type). Moreover, interface engineering can be used to control the flow of electrons. All such processes can result in a vital change in the charge transfer phenomenon, which is a fundamental property. Hence, it would be strategically easier to develop more active and stable electrocatalysts for various applications by understanding the electronic structure.^124^

### Functionality

The desirable functionality of the support, linkers, and catalytic molecules is a prerequisite for the overall stability and performance of electrocatalysts. The selection of molecules bearing the appropriate functional groups for covalent attachment to the support material is equally important as the selection of support and linkers, if required, in terms of functionality. The nature of the functionality significantly influences the electrocatalytic activity of the resulting materials. For instance, support functionality can induce charge transfer and adsorption of reactants and therefore can contribute to the enhancement of the activity, selectivity, and stability of electrocatalysts. The native functionality of the graphene support material includes C=C bonds, and oxygen functionality (in the case of rGO) can be used to further bind the organic linker molecule or catalytic molecules on its surface. The presence of conjugated carbon double bonds is the main cause of the high electronic conductivity of graphene and a suitable work function for the interface electron transfer process. The functionality of the linkers can participate in determining the reaction kinetics. It can adjust the mass transfer process as well as the diffusion of reactants and products, amending their binding energies. Specific functional groups on the linkers can improve the stability and prevent stacking, resulting in better dispersibility in different solvent systems. Consequently, it provides uniform deposition on the electrode surface. Importantly, the electron donation and withdrawal nature greatly influences the conductivity of the material by modifying the electron density, which in turn can alter the redox potential of a particular electrochemical reaction. In fact, –N functionality is found to enhance the electrocatalytic activity due to its electron-donating nature.^54^ The introduction of two or more functional groups can result in synergistic effects. Moreover, the functionality of the attached catalytic molecules can impact performance by influencing the electronic structure through the steric environment around the active sites. Moreover, the molecular structure of catalytic molecules is important for their functionality, which is available for cooperation with the support material as well as the electrode surface. Hence, the availability of desirable functional groups on the support, linker, and catalytic molecules can help in modulating the electronic properties and the reactant adsorption and product desorption processes, thus changing the reaction pathways.^125,126^

### Surface Area

A high surface area is a key factor in achieving higher performance in terms of reaction rate and current density due to the accommodation of a large number of active sites and their facile access to the reactive species during the reactions. More reactive species can adsorb at desirable sites at the same time without approachability restrictions due to the availability of various channels. The surface area is crucial as electrochemical reactions occur at the catalyst surface. A high surface area with a desirable pore size distribution can improve the reaction rate by allowing a large number of active sites to participate in electrochemical reactions. Moreover, it can also enhance the mass transfer process by the construction of various pathways with lower resistance. Accordingly, graphene nanomaterials with high surface areas are generally preferred. Importantly, in electrochemical reactions, the electrochemical surface area (ECSA) is calculated to evaluate the real potential of the electrocatalysts for the target reaction. ECSA is basically a measure of the surface area actually accessible to the reactants and is generally determined by using cyclic voltammetry techniques. Moreover, ECSA is also important for comparing the intrinsic electrochemical activity of various catalytic materials. Once the relationship between the electrocatalytic activity and surface area is established, it can assist in designing an efficient electrocatalyst. Hence, we can control the reaction rate by adjusting the surface area to achieve the superior overall performance of the electrochemical reaction.^127,128^

### Dispersibility

The dispersibility of the electrocatalyst is also an important parameter for the stability and fabrication of the working electrode. Highly dispersed materials are easy to coat with electrodes, providing a sleek and uniform layer of the catalytic material with better stability, thus helping to improve the reaction rate. A high degree of dispersion of the electrocatalytic material in the working solvent system can expose more active sites to the reactant molecules than their aggregates due to the comparatively lower surface area, thus obstructing electrochemical reactions. Moreover, good dispersion ensures not only a uniform distribution of active sites but also constructive interactions with the electrode surface that can improve mass transfer. Consequently, high selectivity and improved reaction rates can be achieved by ensuring a high possibility of interaction between the active sites and the reactants. On the other hand, poor dispersibility can result in restacking of the nanosheets of the catalysts. This can limit the number of charge diffusion channels and, thus, restrict the transport of reactants and products. Generally, reduced oxide graphene has high conductivity but poor dispersibility in most of the working solvents. However, their lower solubility restricts their sole applicability as electrocatalysts. In this regard, the chemical functionalization process stands alone to not only enhance the dispersibility of the graphene material but also generate extra adsorption and active sites. Graphene surfaces can react with many organic molecules in many ways, and these organic molecules can be further grown into polymeric units. Such modifications also lead to comparatively higher dispersibility. Most importantly, the dispersibility of the material can also be tuned by selecting different organic molecules to attach to the graphene surface. The functionality of the functionalized organic molecule determines its degree of dispersion in the respective solvents. In addition to functionality, the structure of organic molecules also affects their dispersion. Finally, such covalent functionalization can be used to tailor the dispersibility of materials in different solvents and the stability of active sites against leaching, besides high conductivity. Hence, dispersibility augmentation is also one of the essential factors to maximize HER efficiency.^129,130^

### Stability

Electrocatalysts must be stable for the development of competitive technologies to generate sustainable energy. Stability is directly related to the activity and selectivity and is a deciding factor for the implementation of an electrocatalyst at the commercial level. Unstable electrocatalysts would degrade with time, resulting in poor performance. On the other hand, long-term stability provides economic viability by reducing the operational costs of time-to-time replacement. Consequently, recognizing the factors affecting the stability of electrocatalysts is crucial. However, achieving optimum stability in terms of chemical resistance is a challenge, as it can be affected by the harsh, demanding conditions of electrochemical reactions. Many times, the high activity of an electrocatalyst is masked by its low stability. Deactivation can occur through various ways, including poisoning, dissolution, leaching, and surface reconstruction. Hence, state-of-the-art in situ characterization techniques can be used to investigate the factors responsible for deactivation and thus help design a material (high-entropy oxides and single-atom catalysts) that can exhibit high activity with long-term stability. There are other ways to improve the overall stability of electrocatalysts, such as coating with protective layers and optimizing electrolytes and components (bipolar plates and membranes). Moreover, interface engineering can significantly stabilize the active sites to achieve a high reaction rate and selectivity by restricting undesired species from reaching the active sites.^131,132^

## Molecular Covalently Functionalized Electrocatalysts for the HER

The beauty of covalent functionalization of graphene to fabricate electrocatalysts lies in the suitable choice of functional molecules. Graphene is an excellent choice for the synthesis of well-engineered graphene-based electrocatalysts because of its low cost and environmentally friendly nature, promising conductivity, high active surface area, and chemical stability. However, the activity can be further enhanced by regulating the surface defects and introducing heteroatoms, metal atoms, and organic molecules. The resulting activity will depend on the type of interaction (covalent or noncovalent) between the graphene support and the introduced species. In this section, we have discussed various graphene-based electrocatalysts, emphasizing the role of covalently functionalized graphene in enhancing HER activity.^133^ Covalent organic polymers (COPs) have been attached to graphene via covalent bonds due to their tunable functionality, chemical composition, and porous structures. However, COPs are undesirable owing to their instability and inferior conductivity. The covalent bond provides a bridge for the facile transfer of charge carriers, resulting in superior electrochemical catalysis for the HER. Therefore, the fabrication of COPs with defined chemical functionalities and their attachment to graphene can overcome the limitations associated with pristine graphene materials as well as bare COPs. Recently, A. Wang et al. have developed a novel nanohybrid of GO and polymeric porphyrin derivatives for the HER.^55^ Polymeric porphyrins are a type of COPs with a conjugated porous structure. Based on the experimental results, it was determined that the covalent interaction between GO and porphyrin-based covalent organic polymers developed a synergistic effect that helped to enhance the HER activity. Moreover, covalent interactions also facilitate the charge transfer process. The stepwise synthesis of the nanohybrid is shown in Figure 4. In this method, first, tetrakis­(4-aminophenyl)­porphyrin (TPP) was reacted with terephthaloyl chloride in the presence of potassium carbonate (K_2_CO_3_) using dimethylformamide (DMF) to construct polymeric porphyrins (TPPCOP).

The successful immobilization of the synthesized TPPCOP on the surface of graphene oxide was performed through an amidation reaction. The terminal amine groups on the TPPCOP reacted with the carboxyl groups at the edges of graphene oxide in the presence of the dehydrating agent dicyclohexylcarbodiimide (DCC) to form a covalent linkage in the form of amido groups, thus resulting in GO-TPPCOP. The reaction was performed in DMF at 80 °C for 4 days. GO-TPPCOP demonstrated excellent HER performance, with a lower overpotential of 376 mV at 10 mA cm^–2^ than their individual peers (TPP, TPPCO, and GO). These results show that not only the polymerization of porphyrin derivatives but also their subsequent immobilization onto the surface of graphene oxide enhanced the HER activity and durability owing to the rigid porous structure and better conductivity. Mechanistically, the involvement of GO not only enhances the electrical conductivity but also acts as an electron acceptor, thus facilitating charge transfer. Moreover, the exposed N-sites and a porous nature also served as favorable factors to reduce the protons effectively. These findings have validated the crucial role of covalent linkages to further enhance the HER activity. The same research group has further utilized zinc phthalocyanines as building blocks to synthesize the metallophthalocyanine-based covalent organic polymeric units (PcP) and their subsequent covalent functionalization onto the surface of GO.^56^ However, *p*-phthalaldehyde and terephthaloyl chloride were used as two different linkers in this process to construct two different covalent organic polymers (PcP 1 and PcP 2), forming Schiff base and amido bonds, respectively, within the polymeric units (Figure 5). Both PcP 1 and PcP 2 were later attached to graphene oxide through an amidation reaction, resulting in two different nanohybrids (GO-PcP 1 and GO-PcP 2).

**Figure 5:**
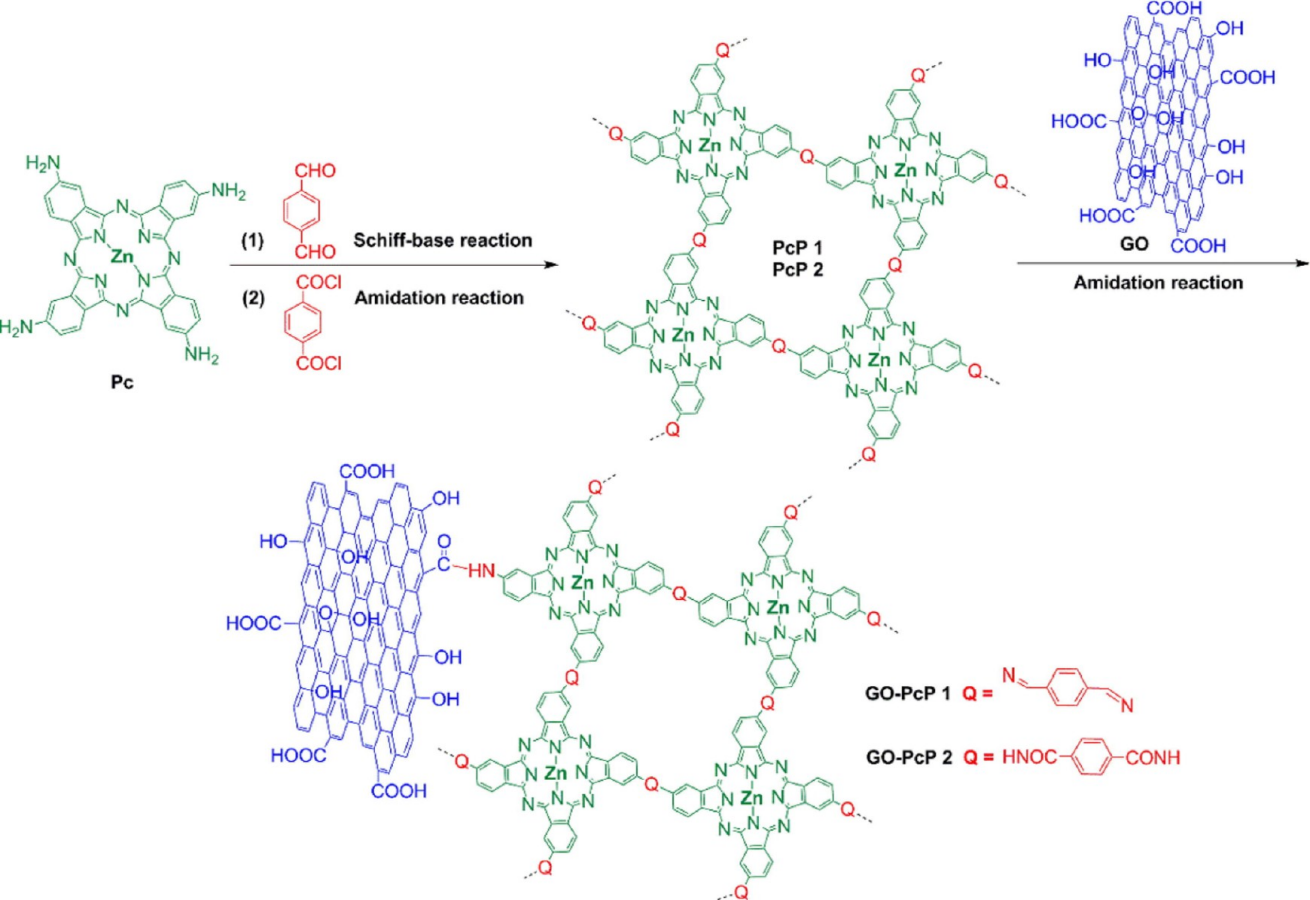
Schematic illustration of the preparation of GO-PcP 1 and GO-PcP 2. Adapted with permission from ref 56. Copyright 2019, Elsevier.

The intention of using two different linkers was to investigate their role in improving the overall efficiency of the resulting nanohybrids. GO-PcP 2 demonstrated superior HER activity in terms of lower overpotentials under acidic conditions compared to other individuals tested in the same study. These results suggest that structural factors, including the type of covalent linkage within the polymeric units, are important to determine the electroactivity of the electrocatalysts for the HER. Moreover, this significant enhancement in performance was also linked to the facile charge transfer between ZnPc-COPs and GO, their higher electrical conductivity, improved surface area, and unique microstructure. The covalent combination of two-dimensional π-conjugated carbon materials with organic molecules such as porphyrins has infused the intrinsic characteristic electrochemical properties of individual molecules to promote the HER. Moreover, their activity can be further tuned for targeted electrochemical reactions. In fact, porphyrin rings have been functionalized with different functional groups, such as –NH_2_, −COOH, and −SO_3_H, to improve the reaction rate under diverse reaction conditions. Notably, the nature of the functional groups, such as electron-donating or electron-withdrawing, and their substituted positions at the porphyrin ring can change their redox reactivity. Similarly, the electronic properties of pi-conjugated carbon materials can be altered by generating defects or intentional functionalization. Accordingly, chemical covalent functionalization, which is a nondestructive approach, can produce heterogeneous, stable, and efficient electrocatalysts that can be sustained in different reaction media. Huynh and co-workers successfully grafted porphyrin derivatives (5,10,15,20-tetrakis­(4-aminophenyl)-21*H*,23*H*-porphine) indicated as a-Por onto the surface of highly oriented pyrolytic graphite (HOPG) and CVD grown graphene (Figure 6).^134^ First, they converted a-Por molecules bearing amine groups into the diazonium radical, a graftable form, (g-Por). a-Por was reacted with NaNO_2_ under acidic conditions for the in situ generation of g-Por diazonium cation, followed by an electrochemical reaction to form g-Por diazonium radical. These were, in turn, successfully grafted on graphitic surfaces via forming C–C bonds through diazotization reactions, yielding a novel 2D hybrid material (g-Por/HOPG). Based on the data, g-Por molecules were not only involved in covalent attachment to the support but also in the formation of a multilayered thin film by reacting with the already attached porphyrin molecules. Basically, the whole process involves the in situ generation of g-Por cations and their electrochemical reduction to produce g-Por radicals, which finally attach directly either to the graphitic support or to pregrafted g-Por molecules, establishing C–C and/or azo (–N=N–) bonding (Figure 6).

**Figure 6:**
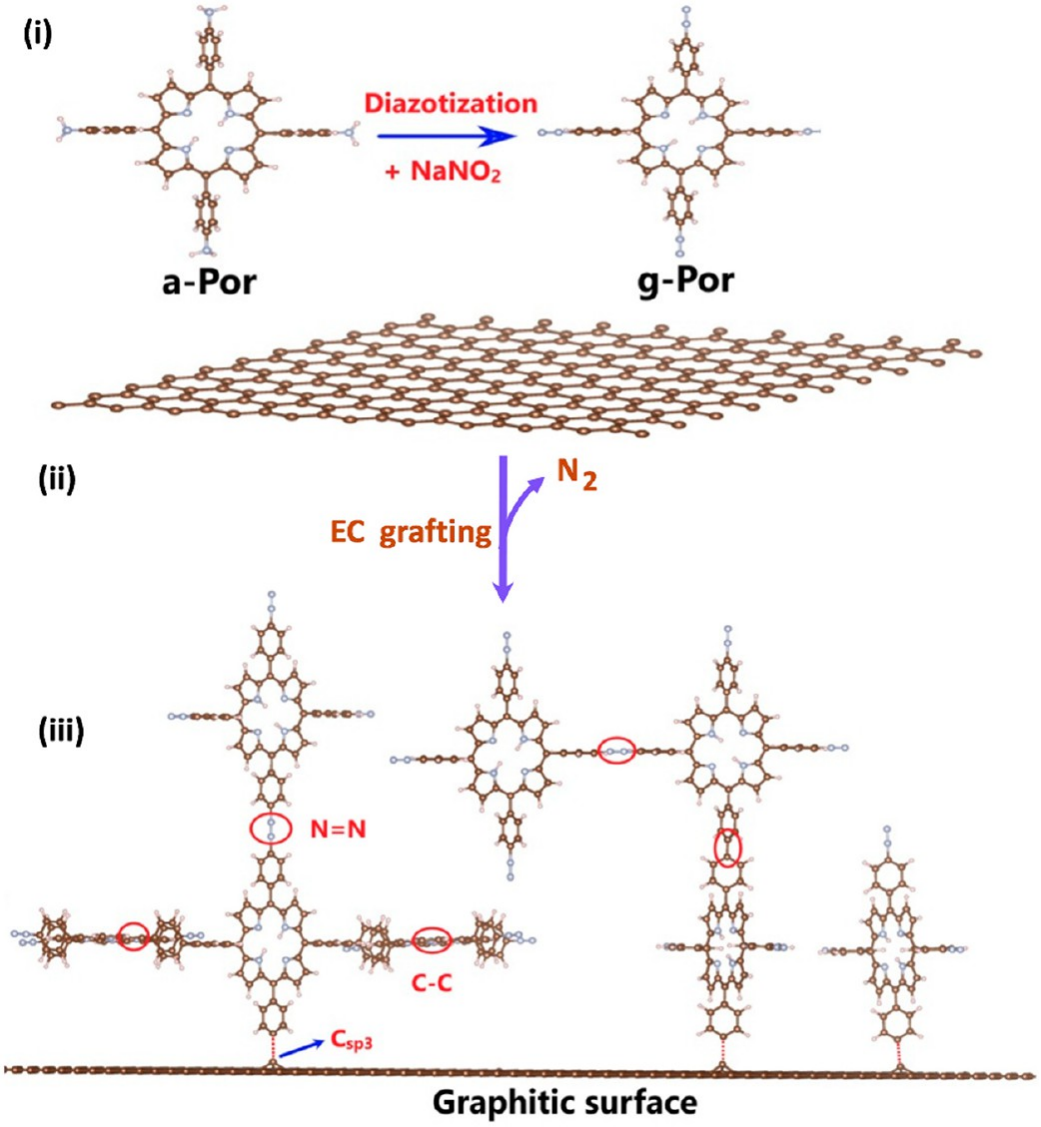
Stepwise formation of the g-Por/HOPG electrocatalyst. (i) Diazonium cation formation; (ii) Reduction of cations to radicals; (iii) Covalent grafting. Part of this figure is adapted with permission from ref 134. Copyright 2024, MDPI.

Strategically, a covalent molecular-anchoring strategy has been employed to improve the HER performance and stability in an acidic medium. Electrochemical experiments revealed that g-Por/HOPG exhibited superior HER activity than bare HOPG, sustaining a current density of 3.0 mA cm^–2^ at 0.39 (V) and 0.75 (V) vs RHE, respectively. Moreover, the g-Por/HOPG fabricated electrode attained an initial onset overpotential of 0.25 V and a current density of 10.0 mA cm^–2^ at an overpotential of 0.53 V vs RHE. This enhancement in the HER activity has been attributed to the formation of interspaced layers while incorporating g-Por molecules, building a vertical structure, and forming a thin layer via covalent bonding. The interspaced layers allow facile access to the imine groups within the structure, thus driving the HER more efficiently. Graphene aerogels (GA) are another interesting material that is generally synthesized via a sol–gel process using a gelling agent, followed by liquid extraction, thus forming a cross-linked high surface area macroporous 3D structure with low density and high electrical conductivity. Accordingly, the application of graphene aerogel-based materials has increased in electrochemical applications, including the HER, owing to the aforementioned properties.

Moreover, the hybrids of GA and COFs have also been synthesized by utilizing the high surface area conductive platform of graphene aerogels to grow stable COFs for further enhancement in the number of active sites and mass transfer. Furthermore, the covalent interaction between graphene aerogels and COFs can further improve the performance of the resulting heterostructure due to the developed synergistic effect and effective mass transfer. However, synthesizing low-density graphene aerogel and growing COFs with desirable functionality without collapsing the nanostructure is challenging. Zhiya Wang et al. have developed a method for the in situ growth of COFs on the surface of GA to fabricate a hybrid of COF/graphene aerogel and investigated its electrocatalytic activity for overall water-splitting reactions.^77^ First, GA was synthesized by mixing GO and l-ascorbic acid and heated at 95 °C without stirring for 5 h to obtain the reduced hydrogel, which was soaked in an alcohol aqueous solution for 2 days and later freeze-dried. GA was then first aminated (GA-NH_2_) using 3-aminopropyltriethoxysilane to grow covalently bonded COFs (GA@Bpy-COF) using 1,3,5-benzenetricarboxaldehyde (TFB) and 5,5′-diamino-2,2′-bipyridine (Bpy) via step-growth polymerization reaction, maintaining the conductive nature and 3D hierarchical porous structures. The Co ions were later complexed with the bipyridine sites of the COFs by soaking GA@Bpy-COF in a methanolic cobalt acetate solution to yield GA@Bpy-COF-Co, which was investigated as an electrocatalyst. The electrochemical results of a comparative study of electrocatalysts prepared and applied for the HER under alkaline conditions revealed that GA@Bpy-COF-Co exhibited a lower overpotential of 275 mV than Bpy-COF-Co (440 mV) and GA@Bpy-COF (710 mV) at a current density of 10 mA cm^–2^ with significant stability. This could be due to the greater electron mobilization and easy access to redox-active sites owing to the porous nature. The fruitful outcome of the preserved electronic and mechanical properties and good coordination of Co ions was clearly reflected in the enhancement of the electrocatalytic activity.

GQDs are mimic fragments of GO with 2–20 nm size and have been metal-free electrocatalysts for water splitting in several studies due to their unique properties associated with the quantum effect. However, chemical functionalization with other molecules can further improve activity by creating additional active sites. In this context, Park et al. have covalently integrated GQDs with ethylene diamine (EDA) molecules, establishing an amido linkage.^135^ Carboxylic groups available on the edges of GQD, synthesized via hydrothermal treatment of GO, were reacted with EDA through an amide coupling reaction to obtain GQDs functionalized with EDA (GQD-EDA). Functionalization was performed using different amounts of EDA under similar reaction conditions to achieve optimal loading. The electrochemical results indicate that the sample GQD-5EDA synthesized by using 0.5 mL of EDA demonstrated superior performance over its contemporaries. Functionalized amine groups acted as water dissociation sites and contributed to efficient charge separation, which in turn subsequently improved the HER activity. Moreover, the performance of the functionalized GQDs in terms of current density and Tafel slope values was found to contradict the activity trend of bare GQDs when the pH of the reaction medium was increased. The lower value of the Tafel slope in the alkaline medium suggested that the functionalized GQDs performed better than the non-functionalized GQDs due to the faster water dissociation step. Hence, the electrochemical results confirmed the role of the functionalization of GQDs with EDA in facilitating the overall reaction rate.

During the HER, H_2_ is produced by the combination of two protons and two electrons at the cathode during the water-splitting reaction. An effective catalyst is the one that allows the electrochemical reaction to proceed at a high rate with a lower onset potential. Molecular catalysts are beneficial because of their precise design and tuning of the chemical nature with the help of ligand modification. However, their homogeneous nature and comparatively low conductivity restrict their potential. Therefore, the covalent attachment of these molecular complexes to the surface of some conductive supports, such as graphene oxide and its derivatives, contributes to the field of electrochemical reactions. The immobilized molecular complex demonstrates some synergetic effect and requires a comparatively smaller amount of the catalyst due to the combined properties of the individual components, limiting cross-contamination between half-cell reactions. Larson et al. have designed an amine-modified cobalt complex, [cobalt bis­(benzylammoniumdithiolate)]^+^, by precuring the amine-substituted benzene dithiol ligand.^82^ The amine-modified cobalt complex reacted with the native epoxide groups of the GO dispersed in DMF at 65 °C for 24 h, forming a covalent linkage. The GO-bonded [cobalt bis­(benzylammoniumdithiolate)]^+^ complex (GO-1) was then drop-casted on a glassy carbon electrode (GCE) for the in situ electrochemical reduction process to obtain reduced graphene oxide-functionalized covalent complex, RGO-1 (Figure 7).

**Figure 7:**
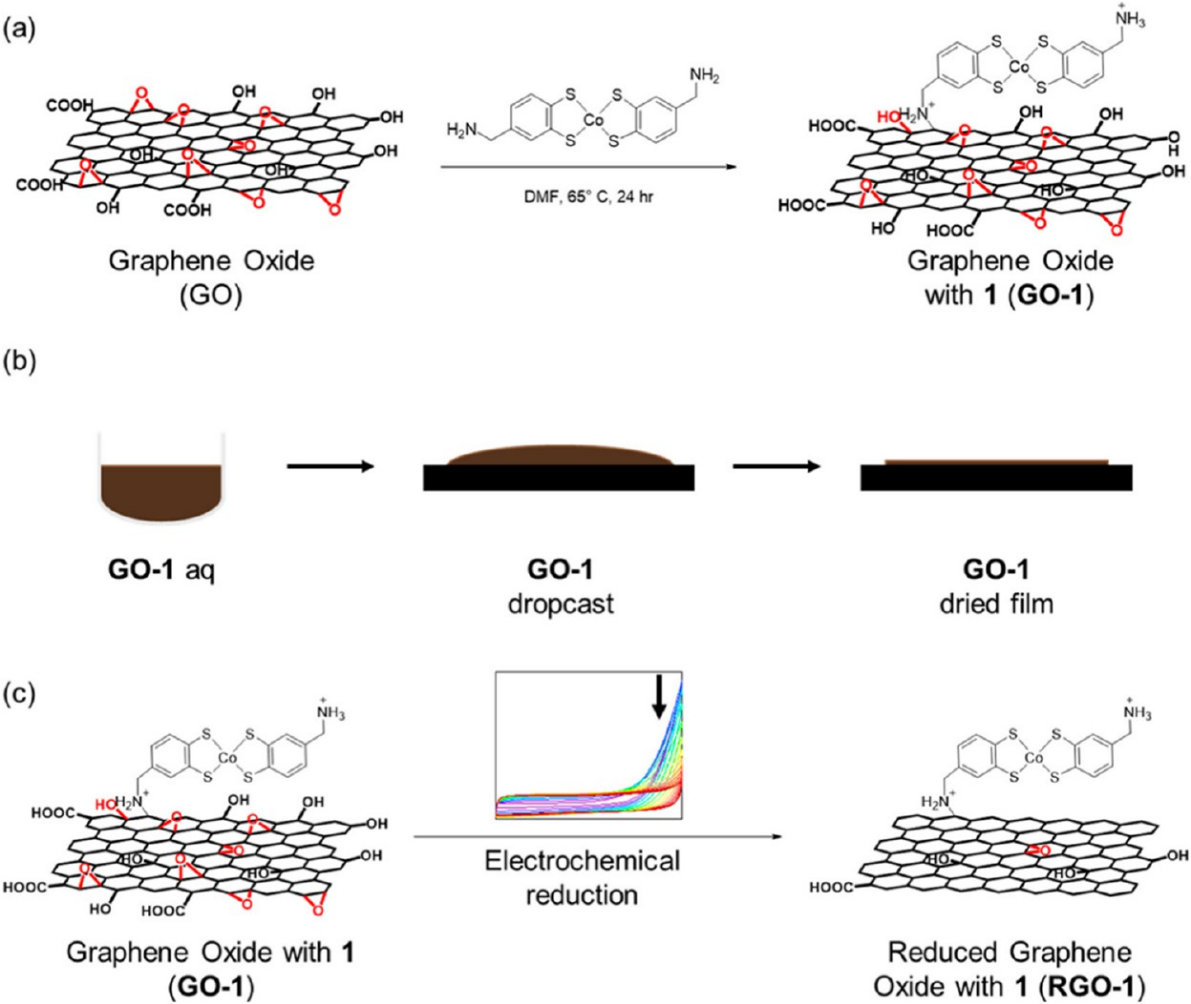
(a) Covalent attachment of 1 to GO to form GO-1; (b) drop-casting of the aqueous suspension of GO-1 onto an electrode surface; and (c) electrochemical GO reduction to RGO. Adapted with permission from ref 82. Copyright 2023, American Chemical Society.

Cyclic voltammetry (CV) was performed to explore the electrocatalytic activity for the HER in an acidic medium. The fabricated electrode with RGO-1 displayed great catalytic activity for HER, resulting in a higher turnover frequency (TOF) (1000 s^–1^ at pH 0) and an overpotential of 273 V at pH 3, while maintaining high Faradaic efficiency (97%). Some disruption in the CV is also observed in the form of a lower current because of the hindering of the access to the electrode surface due to the formation of a significant amount of H_2_ gas bubbles. This could have lowered the electrochemically active surface area. Importantly, the synthesized catalyst has demonstrated robust activity even in ambient air for longer processes, delivering a FE of 79%. These results further highlight the importance of covalent attachment of molecular catalysts onto the graphene oxide surface to improve not only the HER activity but also their stability for long-term operations. In another study, oleyl amine was covalently functionalized onto the graphene oxide/Cu_2_ZnSnS_4_ composite (GO/CZTS) to improve the rate of the water reduction reaction.^136^ The electrocatalytic results have proved the apparent involvement of functionalized oleyl amine molecules, an electron-donating group, in improving the electrical conductivity of graphene and thus the electrocatalytic activity. The authors have also highlighted the importance of selecting oleyl amine over other amines in terms of its high viscosity, boiling point, and reducing and coordinating nature. The catalyst was produced by a three-step process involving the conversion of GO into GO–COCl in the presence of SOCl_2_ + DMF under refluxing conditions for 24 h. These nanosheets were further mixed in a solution of oleyl amines and DMF and sonicated. In the last step, Cu_2_ZnSnS_4_ (CZTS) nanoparticles were added to the resulting dispersion and sonicated for another 2 h at room temperature. The obtained solid material was washed and annealed at 170 °C for 1 h to yield OAm-GO/CZTS (Figure 8). During the thermal treatment, oleyl amine reacted with the functionality of GO, affording oleyl amine-functionalized GO/CZTS (OAm-GO/CZTS). In this catalyst, CZTS nanoparticles are wrapped with amine-functionalized GO nanosheets, creating an interface between oleyl amine-functionalized graphene and CZTS nanoparticles, thus facilitating the charge transfer process, which led to excellent electrocatalytic activity toward the HER. The electrochemical data in the form of a lower overpotential of 47 mV and a lower Tafel slope value of 64 mV dec^–1^ at 10 mA cm^–2^ for the OAm-GO/CZTS electrocatalyst demonstrate its excellent electrocatalytic activity for the HER. This improved performance was attributed to the introduction of amine groups on the GO surface through covalent functionalization, which enabled synergy between the reactivity.

**Figure 8:**
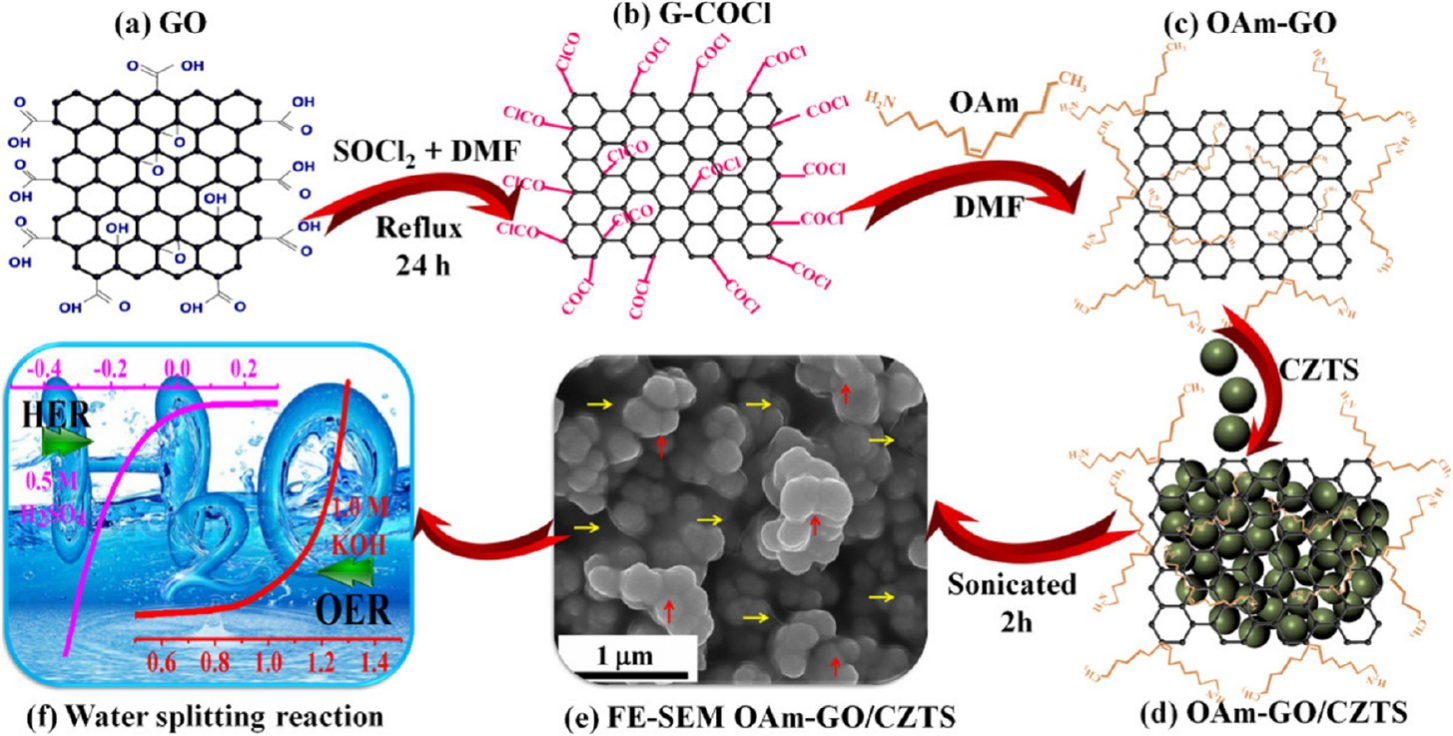
Schematic illustration of the synthesis of OAm-GO/CZTS composites. (a) GO; (b) G-COCl; (c) OAm-GO; (d) OAm-GO/CZTS; (e) FE-SEM OAm-GO/CZTS; (f) Water splitting reaction. Adapted with permission from ref 136. Copyright 2019, American Chemical Society.

Kayan et al. have developed two methods for the preparation of well-dispersed palladium nanoparticles onto the imine-functionalized GO.^137^ First, GO is functionalized with 3-aminopropyltriethoxysilane, followed by the reaction with 2-pyridinecarboxaldehyde, generating a Schiff base functional group (GO/APTES/Scb) (Figure 9). Pd nanoparticles were implanted onto the surface of this composite using two different approaches. The first approach involved the direct electrochemical deposition of Pd nanoparticles to yield rGO/APTES/Scb/Pd Nc1. The other approach involved the initial complexing of Pd^2+^ with the Schiff base functional groups of GO/APTES/Scb, followed by their deposition on the glassy carbon electrode surface for electrochemical reduction, resulting in rGO/APTES/Scb/Pd Nc2. Material characterization and electrochemical results have shown that covalent molecular functionalization is a necessary step to enhance the HER by providing extra sites for H adsorption and effective and uniform Pd binding. Moreover, higher ECSA values indicate the easy availability and accessibility of more catalytically active sites. The deposition of Pd nanoparticles further improved the overall performance significantly by reducing the intrinsic resistance. In fact, the Tafel slope values for both rGO/APTES/Scb/Pd Nc1 (138 mV dec^–1^) and rGO/APTES/Scb/Pd Nc2 (129 mV dec^–1^) were found to be lower than those for the other materials as well as the reported rGO/Pd electrocatalysts. Notably, rGO/APTES/Scb/Pd Nc2 exhibited the lowest Tafel slope value and onset potential of −190 mV, suggesting that the metal complexing step promoted a densely populated distribution and higher loading of Pd nanoparticles. Based on these results, we can conclude that covalent functionalization plays an unprecedented role in not only the distribution and deposition of Pd nanoparticles but also in the electrocatalytic activity for the HER by establishing synergy between Pd nanoparticles and functionalized rGO nanosheets. Moreover, the functionalization process may also optimize the surface area and electrocatalytic properties of Pd nanoparticles as well as the durability of the catalyst.

**Figure 9:**
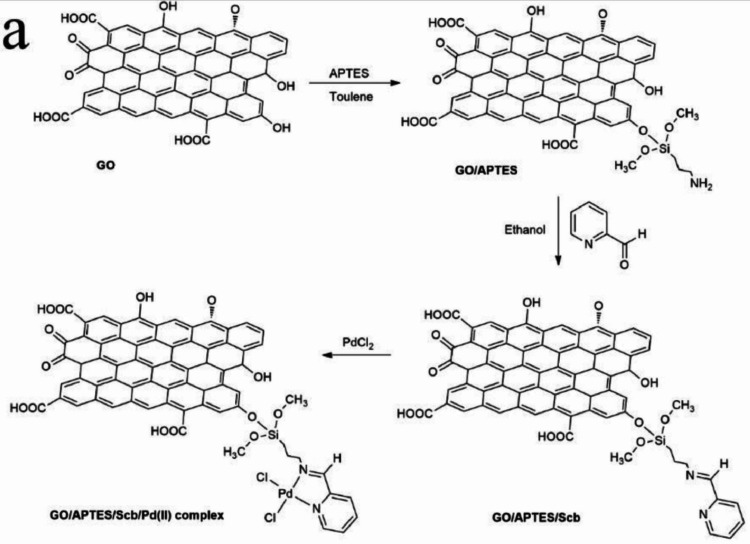
(a) Synthesis scheme of the GO/APTES/Scb/Pd­(II) complex. Adapted with permission from ref 137. Copyright, 2022 Elsevier.

Morphological changes in the nanoparticles can do wonders in achieving optimal reaction efficiency. Different morphologies can exhibit different catalytic activities and stabilities due to the presence of distinctive crystal defects in the form of step, terrace, and edge-site atoms. Therefore, the synthesis of nanomaterials with controlled morphologies is of great interest nowadays. In fact, morphological changes can be achieved by adjusting the synthesis reaction conditions using some shape-directing agents and support material properties. An interesting study involves the use of poly­(vinylpyrrolidone) (PVP) molecules to functionalize graphene oxide nanosheets and then successively utilize the nucleation of Pd nanocrystals (Pd NCs) in three different morphologies. The growth of Pd NCs was achieved by a hydrothermal method, and their morphologies were controlled by varying the reaction time. For the preparation of PVP-functionalized graphene, GO and PVP were stirred together for 10 h, followed by mixing with the required amount of ammonia and hydrazine while stirring for another 45 min at 80 °C. Next, PVP-functionalized graphene dispersed in water was uniformly mixed with PdCl_2_ and ascorbic acid and transferred to an autoclave to heat the resulting homogeneous mixture at 110 °C for different time intervals using a muffle furnace (Figure 10). The duration of the hydrothermal process was found to be crucial for determining the morphology of the Pd NCs. Three different morphologies, Pd-triangle, Pd-nanocubes, and Pd-nanodendrites, were achieved when heated for 8, 10, and 12 h, respectively.^57^

**Figure 10:**
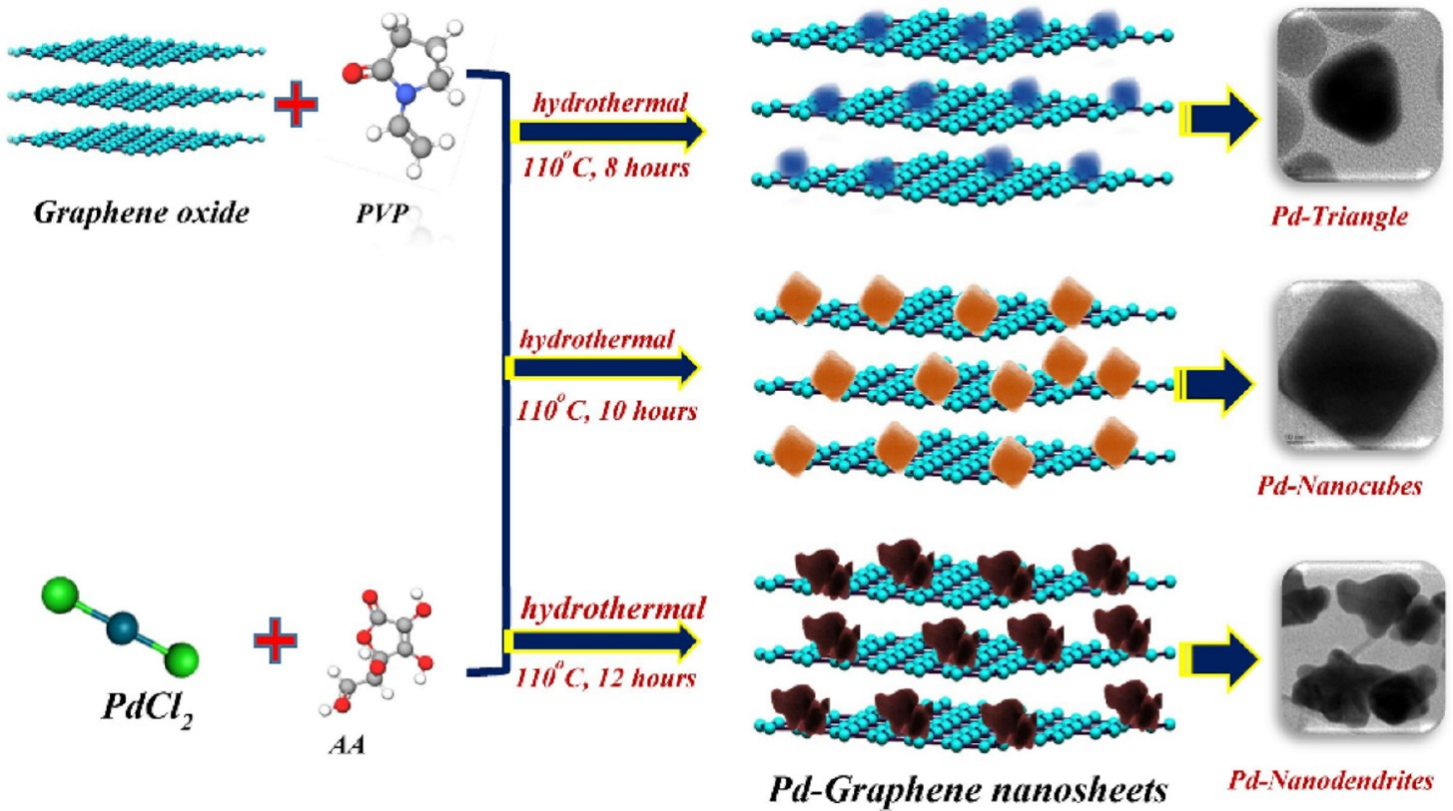
Schematic preparation process of Pd NCs/GNS with different morphologies. Adapted with permission from ref 57. Copyright 2021 Elsevier.

Notably, all the corresponding electrocatalysts, Pd-triangle/GNS, Pd-nanocubes/GNS, and Pd-nanodendrites/GNS, performed differently in terms of loading, distribution, and morphologies of Pd NCs and electrocatalytic activity for the HER. In particular, the Pd-nanodendrite/GNS performed effectively for the HER process owing to the high density of active sites in the form of crystal defects (edge-site atoms, steps, and terraces). Moreover, the high surface area of the branched structure might facilitate the adsorption and stimulation of the reactant molecules. Based on electrochemical experiments, Pd-nanodendrites/GNS required a comparatively lower overpotential of 39.6 mV than Pd-nanocubes/GNS and Pd-triangle/GNS to achieve a current density of 10 mA cm^–2^ in basic medium. Moreover, the smaller Tafel slope of 29.7 mV dec^–1^ indicates significant improvement in the catalytic kinetics of HER. The graphene layers can facilitate the transportation of electrons to the uniformly distributed active sites, thus reducing the Hads (adsorbed hydrogen ions) and liberating H_2_. It was concluded that not only the growth of Pd nanocrystals on the functionalized graphene nanosheets but also their desirable morphology is necessary to enhance the electrocatalytic activity for HER. This study is a good example of designing electrocatalysts incorporating a covalent functionalization approach for better results in terms of loading and controlling the morphology of MNPs.

Genuinely, the covalent functionalization approach is also desirable for metal-free electrocatalyst synthesis. The functionalization of graphitic 2D materials with organic molecules can do wonders in fulfilling the requirement of environmental friendliness. Subsequently, it is desirable to investigate the activity of different organic molecules and polymers rather than the activity of different metals. Kapuria et al. have demonstrated a metal-free approach to boost the electrocatalytic activity of reduced graphene oxide-based electrocatalysts by the covalent functionalization with polysulfide groups.^70^ The different degree of loading of polysulfide molecules was achieved via the thermal condensation method to determine their effect on the enhancement of activity for the HER. This synthesis technique provides a uniform growth of polysulfides without destroying the core structure of GO. The enhanced electrochemical activity was found to be the result of the inherent properties of individual polysulfides and the graphitic framework. The nonbonding electrons in polysulfides and the conductive nature of the graphene structure provide a high number of H-adsorption sites and facilitate facile electron transfer, respectively. The catalyst with the highest polysulfide loading demonstrated the best activity for the HER (overpotential of 254 mV, onset potential of 97 mV, and current density of 10 mA/cm^2^) without showing a significant loss in current density even after 24 h. This appreciable enhancement in electrochemical activity was attributed to the well-exfoliated nature of the covalently functionalized rGO with polysulfide units, facilitating easy H^+^ ion transport and access to two different types of S-active sites. S– linked to the C– of graphene and S– linked to the S– of polysulfides provide delocalized and localized electron clouds, respectively, facilitating easy adsorption and desorption of the H^+^ ions. Hence, this study further validates the importance of desirable covalent functionalization to restrict the stacking of graphene nanosheets and tune the active sites for better HER activity. On the other hand, Puthirath et al. have utilized plasma-based dry functionalization techniques to functionalize graphene and demonstrated the effect of various functional groups on the productivity of the HER.^59^ This functionalization method overcame the drawbacks associated with the inertness of graphene layers toward functionalization with other molecules and materials. Plasma-assisted initial functionalization opens the way to further improving and regulating the extent of functionalization. Functional groups such as −COOH, –O, –NH_2_, –N, and –F were introduced using formic acid, O_2_, –NH_3_, N_2_, and CF_4_ vapor, respectively, to the edges and on the planes of the graphene surface without causing significant damage to the graphene skeleton and generating no harmful waste material. The plasma-assisted change in the functional group attachment to the graphene surface improved their dispersibility and compatibility with various solvents. Moreover, it also affects the activity toward the HER under acidic conditions. In fact, the −COOH and O^+^ functionalized samples demonstrated superior catalytic activity, and the NH_2_, N_2_, and F– functionalized materials showed inferior activity compared to bare graphene. The lowest charge transfer resistance was measured for −COOH functionalized materials, GNP–COOH, among all the materials, and was found to be stable over a time of ∼18 h. These results support the assumption that functionalization with appropriate molecules can significantly enhance the electrocatalytic activity. It is well-known that the electron-donating and -withdrawing nature of each functional group imparts some characteristic properties to the support materials that can affect the catalytic activities of the resulting materials. In this context, Deng and co-workers have examined the role of electron-donating functional groups in the HER by introducing amine groups to graphene nanosheets.^60^ In this process, graphene sheets were functionalized with amine using ammonia, followed by ball milling using KOH to produce highly torn amine-functionalized nitrogen-doped graphene (HT-AFNG). HT-AFNG demonstrated better electrocatalytic activity for the HER in acidic solution owing to N-doping and amine functional groups.

The onset potential and overpotential of metal-free HT-AFNG for the HER were calculated to be 100 and 350 mV, respectively, to attain a current density of 10 mA cm^–2^. These values are smaller than those of the other doped carbonaceous materials. Based on DFT calculations, the electronic structure of nitrogen-doped graphene can be altered by inducing polarity through the electron transfer phenomenon. Subsequently, the availability of amine groups can significantly contribute to achieving a higher catalytic activity for the HER. The electrocatalytic activity of HT-AFNG was compared with that of NG by calculating the Δ*G*_H*_ values for *H* adsorption on active sites with and without amine groups. The presence of amine groups can reduce the Δ*G*_H*_ value and facilitate electron transfer. Moreover, the Fermi levels of –NH_2_ functionalized pyridinic-N and pyrrolic-N were calculated to be more positive than those of pyridinic-N and pyrrolic-N, respectively, thus indicating their higher ability to capture induced electrons. Moreover, the stability of HT-AFNG was determined to be higher than NG, further highlighting the importance of amine groups to improve stability. The highly torn structure of the catalyst itself is also responsible for the high catalytic activity, as it provides easy access to the active sites owing to its porous structure.

The construction of hybrid materials involving graphene is a promising alternative. Bare graphene-based materials tend to restack due to strong van der Waals forces; however, they exhibit high conductivity and high specific surface area. Therefore, coupling with other nanomaterials or molecules is an effective strategy to restrict the stacking of nanosheets and also to preserve high connectivity and accessible surface area. However, the integration of such nanomaterials can be covalent or noncovalent. Based on the reports available, heterostructures with covalent interactions perform better due to facile mass transfer and stability. Chalcogenides are nanomaterials that are coupled with graphene to develop efficient electrocatalysts, mainly due to their exposed edge sites. Molybdenum disulfide (MoS_2_) belongs to the category of transition metal dichalcogenides and has a semiconductive 2H-phase. The nanosheets of layered materials exhibit more characteristics than their bulk materials. Subsequently, the nanosheets of 2H-MoS_2_ can be synthesized via various methods such as hydrothermal, solvothermal, and CVD methods. It is nontoxic, abundant, inexpensive, and has a high surface activity and specific surface area. Consequently, it is an attractive candidate for the development of HER catalysts. However, poor electronic conductivity restricts the achievement of optimal activity. Therefore, the incorporation of MoS_2_ nanosheets with some conductive materials, such as carbon materials, can overcome this limitation. In fact, graphene-based materials have been used to enhance the total conductivity of the resulting catalytic material. However, stacking nanosheets of MoS_2_ and graphene materials may occur due to van der Waals forces, which can limit the access to the active sites on their respective surfaces. The integration of MoS_2_ nanosheets with graphene can result in a high number of active sites and better electronic conductivity. Therefore, such heterostructures have demonstrated a significant improvement in their activity for electrochemical applications. However, precovalent surface functionalization of graphene materials is desirable for the fabrication of hybrid materials due to their ease of charge transfer, which limits their stacking and ability to bind with other nanomaterials. Li et al. have integrated the MoS_2_ nanosheets with the *p*-phenylenediamine (PPD)-functionalized reduced graphene oxide/O-containing carbon nanotubes (rGO/O-MWCNT) through electrostatic and π–π stacking interactions following a one-pot hydrothermal process.^58^ In this method, functionalization with PPD led to the facile growth of MoS_2_ sheets during the hydrothermal process, leading to the highly exposed surface area and allowing easy access to the high number of edge sites. Moreover, the successful construction of the 3D structure improved the mass transfer process. MoS_2_/rGO/PPD/O-MWCNT demonstrated a comparatively lower overpotential and Tafel slope of 48 mV·dec^–1^, high current density (47.6 mA·cm^–2^ at 200 mV), and excellent stability up to 1000 cycles for the HER in acidic solution. However, the performance declined because of the absence of PPD functionalization, showing higher internal and charge transfer resistance. These results are important to validate the active role of PPD covalent functionalization in HER activity by providing additional pathways for fast charge transport.

In addition to the electrostatic or π–π interacted hybrids of MoS_2_ and functionalized graphene materials, a pure covalent interaction utilizing the suitable functional groups present on the surface of the respective MoS_2_ and graphene materials is also a desirable strategy to improve the HER activity. This process can overcome the drawbacks associated with the insignificant electronic conductivity of MoS_2_. Accordingly, these hybrids have shown better activity for the HER due to better interconnectivity through covalent linkages, thus improving electron transfer and access to a high number of active sites. Pramoda et al. have successfully cross-linked the nanosheets of carbon nitride (C_3_N_4_) with MoS_2_ or RGO establishing amide covalent linkages via coupling reaction and investigated their HER activity (Figure 11).^138^ For the construction of the C_3_N_4_–NRGO heterostructure, carbon nitride is reacted with the synthesized NRGO in the presence of *N*-(3-(dimethylamino)­propyl)-*N*′-ethylcarbodiimidehydrochloride, 1-hydroxybenzotriazole, and *N*,*N*-diisopropylethylamine under an inert atmosphere using DMF solvent at room temperature for 48 h. For C_3_N_4_–MoS_2_, carboxylate-MoS_2_ (MoS_2_–CH_2_COOH) was first synthesized by a room temperature reaction with an excess amount of 2-bromoacetic acid for 5 days. MoS_2_–CH_2_COOH was subsequently reacted with C_3_N_4_ following the same method. Interestingly, C_3_N_4_–NRGO and C_3_N_4_–MoS_2_ showed better BET surface areas of 186 and 151 m^2^/g, respectively, than the physical mixture of their components. Moreover, the HER performance of both synthesized heterostructures (C_3_N_4_–NRGO and C_3_N_4_–MoS_2_) was found to be superior to that of individual C_3_N_4_ and NRGO or MoS_2_ and their corresponding physical mixtures.

**Figure 11:**
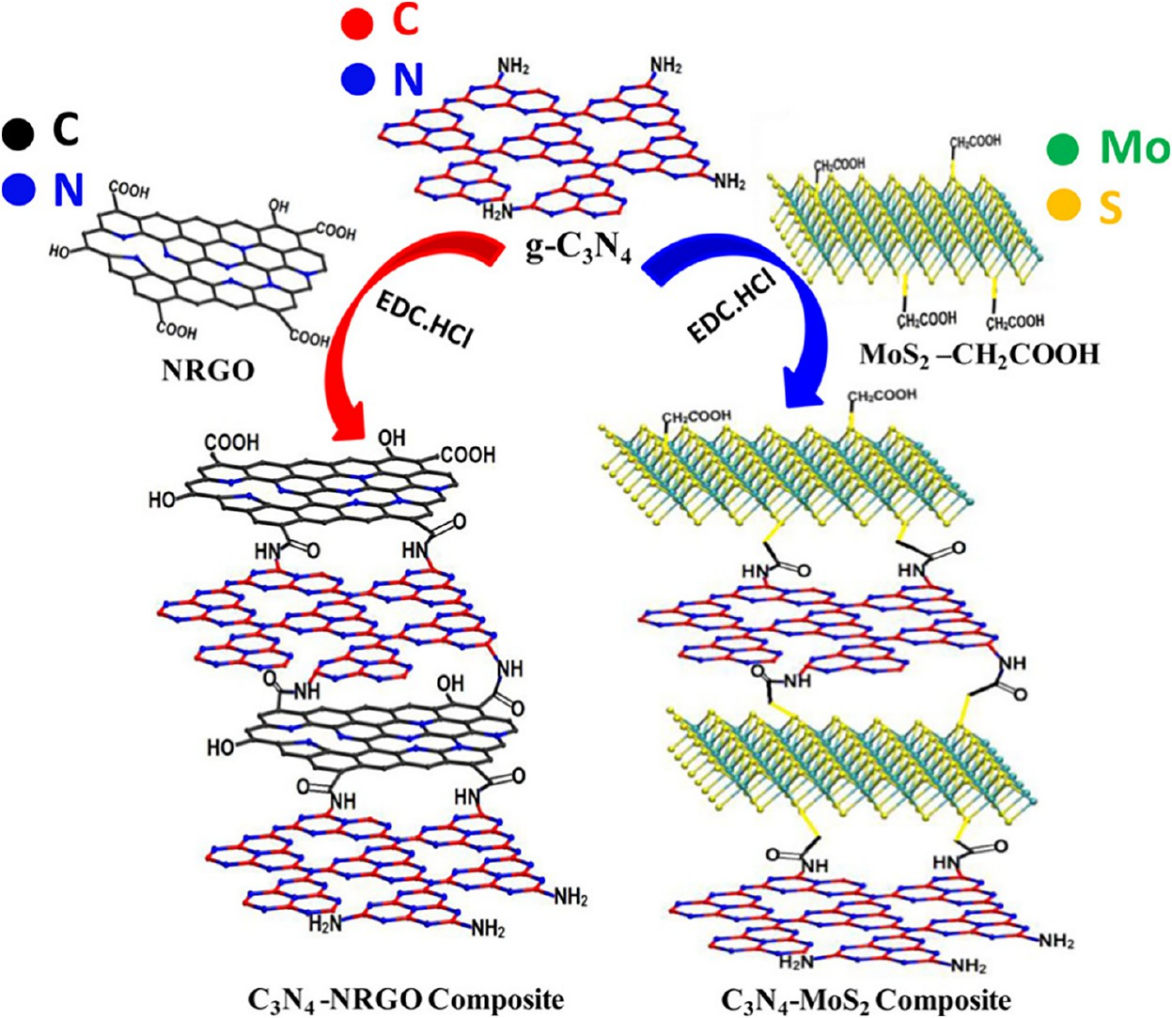
Strategy for covalently bonded C_3_N_4_–NRGO and C_3_N_4_–MoS_2_ composites. Adapted with permission from ref 138. Copyright 2017, American Chemical Society.

The trend in the enhancement of the activity is as follows: covalent bonding > physical mixtures > individual C_3_N_4_. Based on DFT, better orbital overlapping due to the higher planarity of the corresponding layers and facile charge transfer due to good interconnectivity between them appeared to be significant factors responsible for the reduction of the onset potential. The onset potentials for C_3_N_4_–NRGO and C_3_N_4_–MoS_2_ were measured to be −0.36 V and −0.23 V, with Tafel values of 147 and 88 mV/dec, respectively. Moreover, a larger electrode/electrolyte interface area due to the generation of porosity and increased surface area as a result of the cross-linking process also contributed. The findings presented here further document the benefits of covalent bonding in designing future nanocomposites for electrochemical applications. The covalent chemical functionalization strategy has also been utilized to prepare nanocomposites with positively charged molecules to facilitate interactions with nanocomposites of opposite polarity. The same group functionalized reduced graphene oxide and borocarbonitride with poly­(diallyldimethylammonium chloride) (PDDA) to make them positively charged.^103^ These positively charged materials (P.RGO and P.BCN) were then electrostatically connected with negatively charged exfoliated MoS_2_ and MoSe_2_ nanosheets to prepare superlattice-like nanocomposites P.RGO-MoS_2_, P.RGO-MoSe_2_, and P.BCN-MoS_2_ via a simple solution process (Figure 12).

**Figure 12:**
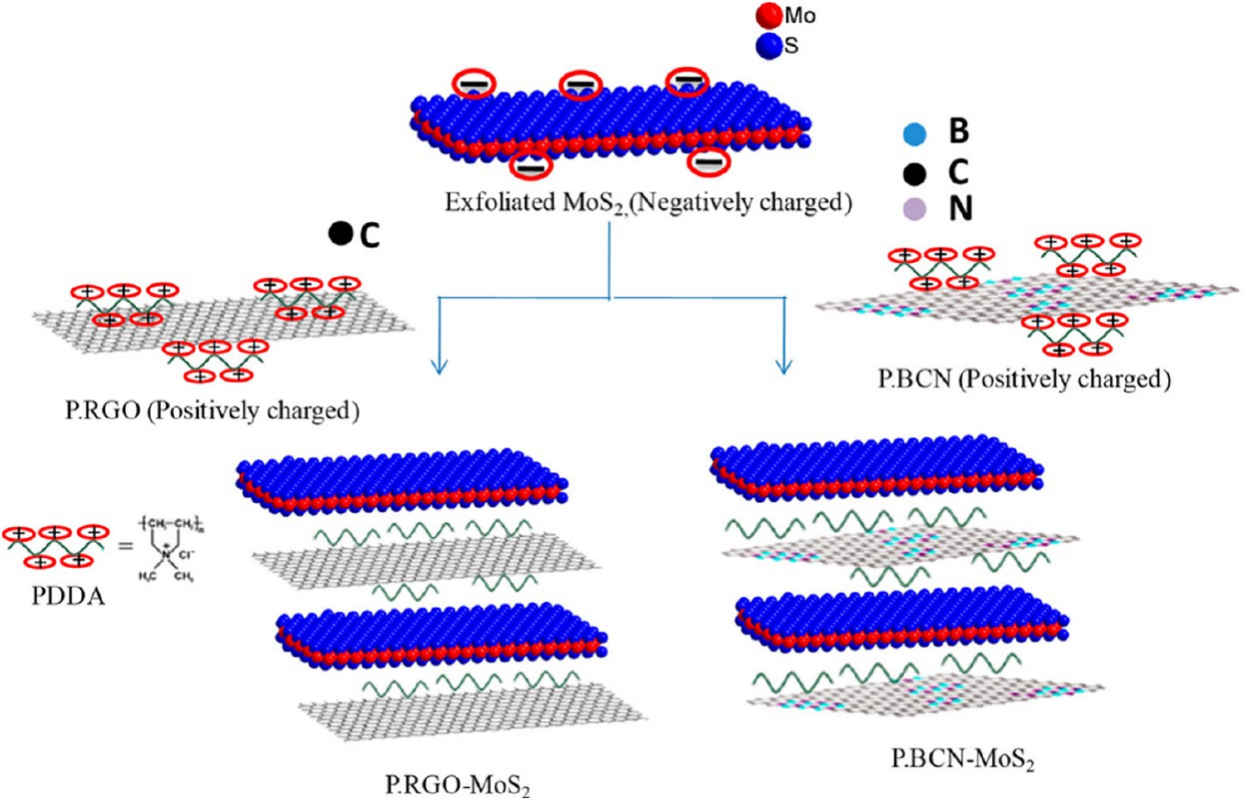
Schematic representation of the synthesis of P.RGO-MoS_2_ and P.BCN-MoS_2_. Adapted with permission from ref 103. Copyright 2020, American Chemical Society.

Nanocomposites with different ratios of contributing materials were also synthesized to investigate the effect of their content ratio on the HER. P.BCN-MoS_2_ nanocomposites with different content ratios were investigated for electrocatalytic HER, and were tested accordingly. The P.BCN-MoS_2_ nanocomposite with a 1:5 content ratio performed better as an electrocatalyst in a H_2_SO_4_ electrolyte, resulting in a lower onset potential of −50 mV compared to the individual P.BCN (−470 mV) and MoS_2_ (−190 mV). The Tafel slope value obtained for P.BCN-MoS_2_ (1:5) was 65 mV dec^–1^, which is closer to that of Pt/C. These results verified the usefulness of electrostatically assembled heterolayers to improve mass transport and to reduce charge transfer resistance. Graphene quantum dots (GQDs) are attractive precursors for electrocatalytic applications owing to their tunable size, high surface area, dispersibility, and availability of ample active sites in terms of functionality. Moreover, they can be further functionalized with suitable molecules to tailor their native properties. In an interesting study, functionalized grapheme quantum dots (NH_2_-GQDs, COOH, GQDs, OH-GQDs, and SO_3_-GQDs) with various electron-donating and electron-withdrawing groups were used to fabricate near-atom-layer 2H-MoS_2_ nanosheets (ALQD) through a hydrothermal process.^139^ In this method, (NH_4_)_6_Mo_7_O_24_·4H_2_O, thiourea, and the required type of functionalized GQDs were mixed thoroughly in distilled water to obtain a homogeneous solution, which was poured and heated in a Teflon-lined stainless-steel autoclave at 200 °C for 20 h to yield the respective ALQD. It was found that electron-withdrawing groups on the GQDs resulted in thinner and more active ALQD by enlarging the layer spacing of 2H-MoS_2_. SO_3_-GQD-assisted fabrication of ALQD-SO_3_ provided a small Tafel slope value of 93.2 mV dec^–1^ and low overpotential (245 mV) to obtain a 10 mA cm^–2^ current density exhibiting superior stability up to 160 h. This data clearly indicated the role of GQD bearing electron-withdrawing groups in improving the reaction rates by regulating the morphology and electronic structure. Electron-withdrawing functionalized GQDs can result in nanosheet-type architectures, depending on the concentration of the groups. On the other hand, the presence of electron-donating groups can result in an aggregated bulk structure, which can lead to sluggish HER kinetics. However, this process suffers from drawbacks such as low quality, production yield, and reduced efficiency due to postprocessing steps, such as crystallization. Hu et al. have also synthesized near-atom-layered MoS_2_ nanosheets (ALMS) and obtained an impressive yield of 63% using an efficient one-step mechanochemical approach utilizing SO_3_-functionalized graphene quantum dots (SO_3_-GQDs) as exfoliation agents.^61^ This ball-milling process required no solvent, thus offering bulk synthesis in a more cost-effective manner and overcoming the associated limitations of previous methods (Figure 13). GQD-assisted synthesized ALMS were found to exhibit a higher specific surface area and outstanding dispersibility in isopropanol solvent.

**Figure 13:**
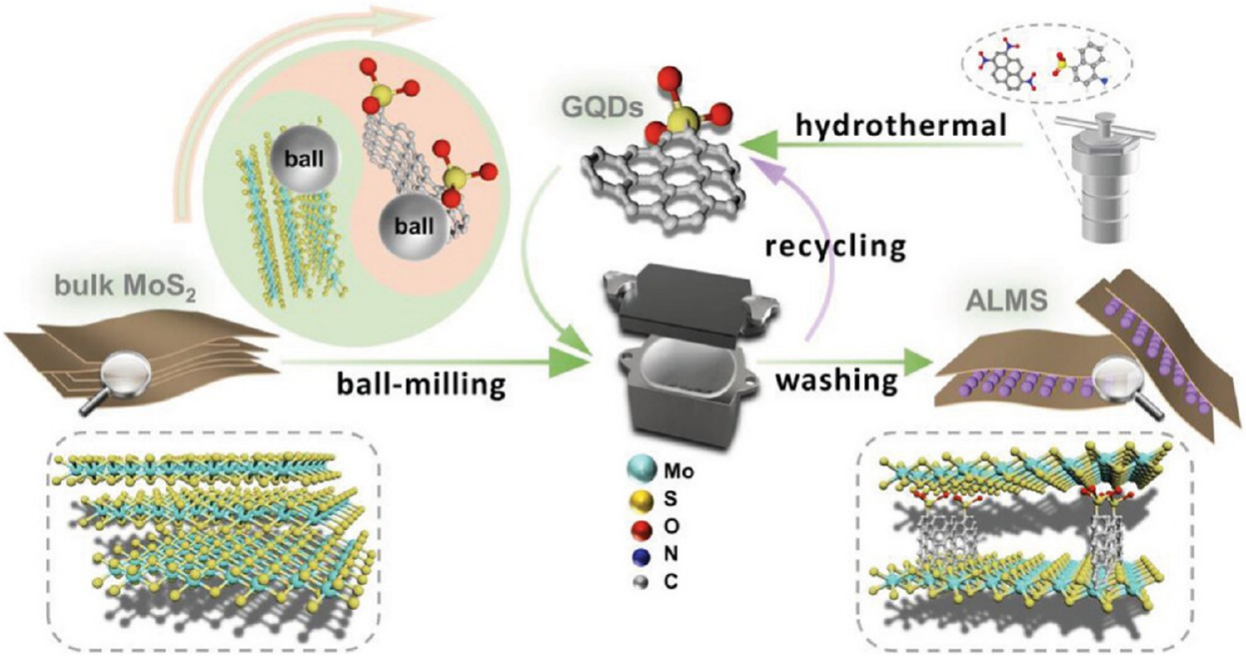
Schematic diagram of ALMS synthesis. Adapted with permission from ref 61. Copyright 2024, Wiley.

The synthesized ALMS, which comprised a combination of vertically aligned 2D MoS_2_ with GQDs, was tested for HER, and it performed excellently to speed up the mass transfer while maintaining long-term stability for nearly 200 h. Moreover, the functionalized GQDs introduced functional groups on the surface of ALMS, increasing the interval distance, thus resulting in a high specific surface area. Thus, exceptional HER activity was shown, attaining a current density of 10 mA cm^–2^ at an overpotential of 270 mV, outperforming bulk MoS_2_. Moreover, the Tafel slope values were calculated to be 253.6, 83.3, and 44.9 mV dec^–1^ for the bulk MoS_2_, ALMS, and Pt/C catalysts, respectively. This study confirmed the paramount role of SO_3_-GQDs in the synthesis of MoS_2_ nanosheets and the sulfonation process to improve not only the stability but also the performance of HER. These results are the best examples to highlight the importance of molecular covalent functionalization even in the field of synthesizing nanomaterials to promote the HER activity.

Electrocatalytic active sites should be properly connected to the support for higher performance. Such connections need to be established to influence the electronic structure by designing catalysts. Wang et al. have used nanostepped TiO_2_ to functionalize graphene quantum dots on its surface through a covalent ester bond formation strategy.^28^ The functionalized GQDs were then used as anchor points for electroactive cobalt phosphide nanoparticles (CoP). In this method, nanosteps were first generated on the TiO_2_ nanowires through hydrothermal treatment at 120 °C with HCl solution for 2 h. The nanostepped TiO_2_ (S-TiO_2_) was reacted with carboxyl-functionalized GQDs, forming an ester linkage to yield GQDs/S-TiO_2_. Finally, CoP nanoparticles were grown through impregnation and phosphidation, resulting in CoP/GQDs/S-TiO_2_ (Figure 14). The synthesized heterostructure materials (CoP/GQD/S-TiO_2_) were tested for their electrocatalytic activity. This systematic arrangement provided highly dispersed active sites and also a more effective path for mass transfer from TiO_2_ to GQDs. The incorporation of GQDs was found to be vital to adjust the electronic structure of CoP, thus resulting in superior HER performance and stability with 6.3 times the mass activity of the counterpart heterostructure without GQDs (CoP/S-TiO_2_). Mechanistically, the electron-withdrawing nature of GQDs due to their conjugated nature facilitates the successful electron transfer from TiO_2_ to Co sites through the Ti–C–P pathway. Consequently, Co centers become electron-rich and can stabilize the alkaline cations (AC^+^), thereby improving HER kinetics. The covalent grafting of GQDs participated in increasing the surface area to promote the homogeneous distribution of small nanoparticles of CoP. Moreover, it also provided long-term durability to CoP/GQDs/S-TiO_2_ for over 100 h through strengthened electronic interactions. This report further highlights the potential of covalent bonding in electronic structure engineering for boosting electrocatalysis (Table 2).

**Figure 14:**
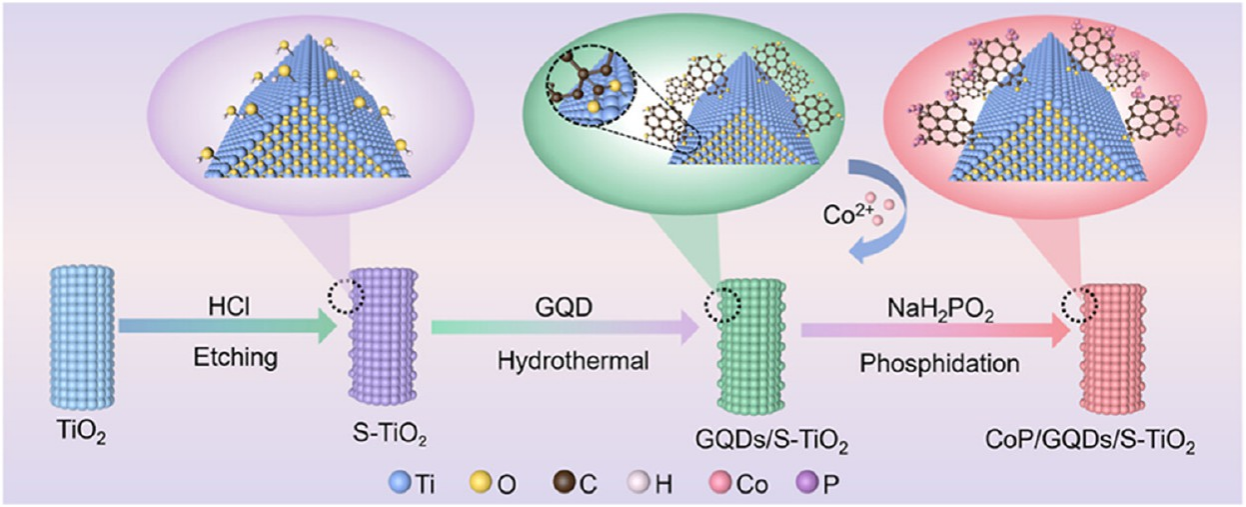
Stepwise synthesis of CoP/GQDs/S-TiO_2_. Adapted with permission from ref 28. Copyright 2025, American Chemical Society.

**Table 2:** Other Graphene-Based Electrocatalysts for the HER

sn	catalysts	synthesis method	HER activity	refs
1.	CoNiP/CoNi nanoparticle/graphene/carbon foams	chemical synthesis	overpotential: 224 mV	^140^
			Tafel value: 85 mV dec^–1^	
			RE: Ag/AgCl	
			CE: graphite rod	
			WE: glassy carbon rotating disk electrode	
			medium: alkaline	
2.	PtRu/graphene	femtosecond laser	overpotential: 15.5 mV (alkaline) and 13.6 mV (acidic)	^141^
			Tafel values: 53.5 mV dec^–1^ (basic) and 24.3 mV dec^–1^ (acidic)	
			RE: Hg/HgO (basic) and Ag/AgCl (acidic)	
			CE: graphite rod	
			WE: glassy carbon electrode	
			medium: alkaline, acidic	
3.	nickel/nitrogenated graphene hybrid	hydrothermal process, silanization, and electrodeposition	overpotential: 950 mV	^67^
			Tafel value: 162 mV dec^–1^	
			RE: SCE	
			CE: Pt foil	
			WE: glassy carbon electrode	
			medium: alkaline	
4.	CoCuFe-LDH/graphene composite	hydrothermal process	overpotential: 380 mV	^142^
			Tafel value: 76.6 mV dec^–1^	
			RE: Hg/HgO	
			CE: Pt counter electrode	
			WE: glassy carbon electrode	
			medium: alkaline	
5.	FeP@3DNG	hydrothermal-phosphidation	overpotential: 76 mV	^143^
			Tafel value: 40.2 mV dec^–1^	
			RE: Ag/AgCl	
			CE: Pt wire	
			WE: glassy carbon electrode	
			medium: alkaline	
6.	NiMo@VG@CC	electrodepositing	overpotential: 70.95 mV	^144^
			Tafel value: 86.77 mV dec^–1^	
			RE: Ag/AgCl	
			CE: graphite rod	
			WE: glassy carbon electrode	
			medium: alkaline	
7.	Co–N/P-FG	ball-milling method, hydrothermal method, and pyrolysis	overpotential: 275 mV	^145^
			Tafel value: 152.88 mV dec^–1^	
			RE: Hg/HgO	
			CE: graphite rod	
			WE: glassy carbon electrode	
			medium: alkaline	
8.	FeCoS_2_/Co_4_S_3_/N-doped graphene composite	hydrothermal synthesis and chemical vapor deposition process	overpotential: 172 mV	^146^
			Tafel value: 67 mV dec^–1^	
			RE: Ag/AgCl	
			CE: Pt plate	
			WE: glassy carbon electrode	
			medium: alkaline	
9.	PtPd/rGO-2	chemical reduction	overpotential: 87.16 mV	^147^
			Tafel value: 18.9 mV dec^–1^	
			RE: SCE	
			CE: graphite rod	
			WE: carbon paper	
			medium: alkaline	
10.	Ni–Pd/rGO	chemical synthesis	overpotential: 63 mV	^148^
			Tafel value: 116 mV dec^–1^	
			RE: Ag/AgCl	
			CE: graphite rod	
			WE: glassy carbon electrode	
			medium: acidic	
11.	N-VGSs@CB/CoP	thermal chemical vapor deposition	overpotential: 138 mV	^35^
			Tafel value: 86 mV dec^–1^	
			RE: Hg/HgO	
			CE: graphite rod	
			WE: glassy carbon electrode	
			medium: alkaline	
12.	N-MoSe_2_/G composites	surface-plasma-induced exfoliation and doping	overpotential: 153 mV	^149^
			Tafel value: 67 mV dec^–1^	
			RE: Ag/AgCl	
			CE: graphite rod	
			WE: glassy carbon electrode	
			medium: alkaline	
13.	G@Co–W–P	hydrothermal pretreatment-mediated self-assembly	overpotential: 13 mV (acidic), 16.1 mV (alkaline)	^150^
			Tafel values: 40.7 mV dec^–1^ (acidic), 61.2 mV dec^–1^ (alkaline)	
			RE: SCE (acidic) and Hg/HgO (alkaline)	
			CE: Pt foil, graphite rod	
			WE: glassy carbon electrode	
			medium: alkaline, acidic	
14.	Mo_2_C-RGO nanocomposite	microwave-assisted solvothermal (MWSV) synthesis	overpotential: 120 mV	^151^
			Tafel value: 85 mV dec^–1^	
			RE: SCE	
			CE: graphite rod	
			WE: glassy carbon electrode	
			medium: acidic	
15.	CoRu_0.1_@Gr	chemical synthesis and in situ growth	overpotential: 34 mV	^152^
			Tafel value: 56 mV dec^–1^	
			RE: SCE	
			CE: graphite rod	
			WE: glassy carbon electrode	
			medium: alkaline	
16.	Pt-3D-LIG	electrochemical deposition	overpotential: 455 mV	^153^
			Tafel value: 73.2 mV dec^–1^	
			RE: Ag/AgCl	
			CE: platinum gauze	
			WE: glassy carbon electrode	
			medium: alkaline	
17.	Ru/Co@OG	chemical synthesis	overpotential: 13 mV	^34^
			Tafel value: 22.8 mV dec^–1^	
			RE: SCE	
			CE: Pt plate	
			WE: glassy carbon electrode	
			medium: alkaline	
18.	Co-SAC	chemical synthesis	overpotential: 230 mV	^33^
			Tafel value: 99 mV dec^–1^	
			RE: Ag/AgCl	
			CE: Pt wire	
			WE: glassy carbon electrode	
			medium: acidic	

## Conclusion

Graphene-based materials have comparatively excellent electronic conductivity and electrochemically active surface area, which makes them suitable for the electrocatalytic applications. Moreover, they are nontoxic and can be prepared in different morphologies by using easily available, inexpensive, and environmentally friendly carbon sources with the help of chemical treatment and/or exfoliation methods (chemical and physical). However, there are always some possibilities to further enhance their activity by applying strategies, such as covalent molecular functionalization, that can augment their properties at different levels. Graphene-based covalent molecular functionalized electrocatalysts have been researched for a long time to develop resourceful technologies to harvest H_2_, a clean source of energy, through HER. These electrocatalysts have a unique set of properties, such as low leaching of activity sites, less agglomeration, faster electron transfer, high specific surface area, and facile access to active sites. In this process, various functional molecules (metal complexes, organic polymers, nanoparticles, and other nanomaterials) bearing active sites are introduced onto the surface of the graphene nanomaterials, establishing covalent bonding. In fact, this strategy has been found to be effective not only to enhance the electrocatalytic performance but also to enhance the stability of the active sites in terms of leaching and has been a centric approach to tailor the properties of electrocatalysts. Various reasons, including the synergy between the active sites and graphene materials, have been proposed to explain the enhanced activity. Accordingly, we have highlighted this synthetic route and its necessity to enhance the HER activity in this review article. We have also thoroughly discussed the role of various precursors such as graphene support, linkers, and functional molecules bearing active sites (metal complexes, organic molecules, polymers, and other nanomaterials that have been attached via covalent bonding). The exploitation of 2D graphene nanomaterials and the mounting of a molecular entity onto their surface through covalent bonding have contributed significantly to improve not only the number of active sites but also their approachability. Moreover, covalent linkages have contributed to facilitate electron transfer between the electrocatalyst and substrate and restrict the stacking of graphene nanosheets, thus allowing the substrates to easily reach the active sites. The introduction of a functionalized moiety can integrate the merits to the parent nanomaterial, affecting its chemical composition and electronic properties, thus stabilizing the active sites and improving the selectivity and reaction rate. Functionalized graphene can show better dispersibility and, therefore, can be deposited on the electrodes more efficiently and interact well with other materials in the case of hybrid catalysts. Moreover, such functionalization can further stabilize the deposition of metal nanoparticles and participate in establishing constructive metal–support interactions. The generated functionality can also help activate the reactants by adsorbing them and thus can actively participate in determining the reaction mechanism. To date, many types of functional moieties, including other nanomaterials (in the case of heterostructures), have been decorated via state-of-the-art synthesis procedures such as diazotization, substitution reaction, silanization, etherification, amidation, hydrothermal, ball milling, plasma-assisted, and cycloaddition reactions. Each individual reaction has its own significance in bringing various innovative properties to the resulting nanocomposites, which in turn can demonstrate potential applications in the HER. Moreover, key aspects of the commercial application of covalently functionalized graphene are to develop low-cost multifunctional electrocatalysts, achieve enhanced processability and scalability, and tailor key properties responsible for better performance. The covalent functionalization process allows the attachment of various molecules (redox-active and proton-conducting groups) to the graphene surface, which can perturb its aromatic arrangement, thus resulting in improved reactivity and selectivity. Hence, a large number of graphene materials with multiple functions can be designed. These materials are effective for electrode and electrolyte membrane cell preparations for supercapacitors and fuel cells, respectively. Moreover, functionalization improves the dispersibility in various solvent systems, including aqueous media, making it suitable for emerging next-generation nanoelectronics applications such as printable electronics. However, the precise control of graphene functionalization to preserve its inherent properties is a challenge.

## Challenges and Future Scope

Bare graphene materials have limitations associated with their high stacking nature, low porosity, and limited active sites. Their systematic modification is a prerequisite for achieving desirable characteristics for the enhancement of electrocatalytic activity. Covalently bonded molecular modifications can be a convenient and suitable approach to introduce functional molecules in the form of molecular complexes, polymers, organic molecules, and other nanomaterials onto graphene nanosheets. Covalent functionalized graphene-based electrocatalysts have been appreciated for their comparative activity toward the HER. However, there are still some gaps to fill in terms of achieving similar or better activity than state-of-the-art Pt/C electrocatalysts. Moreover, challenges mainly related to stability and activity can be addressed by choosing the right direction for the design and synthesis of electrocatalysts. Graphene layers are susceptible to oxidation at high temperatures under humid atmospheric conditions and thus corrode during long-term operations, leading to structural changes. Moreover, the presence of residual functionality, depending on the graphene derivatives, can undergo transformations, altering the catalyst’s electronic structure and properties. The detachment of functional molecules from the surface of graphene can occur under varying reaction conditions, causing unstable dispersion and restacking of nanosheets. Other stability concerns include the deactivation of active species by the reaction of in situ-generated intermediates under surprisingly unfavorable conditions during the reaction.

The stability of graphene-based electrocatalysts can be improved by following various approaches, such as defect engineering, heteroatom doping, interface engineering, functional group incorporation, hybridization, encapsulation, and constructing 3D porous structures. These approaches can provide structural support, prevent corrosion, and create protective structures, thus exhibiting superior catalyst endurance during operation. In addition, electronic properties can be significantly amended that can further boost the electrocatalytic activity. For example, encapsulation and hybridization methods involve the wrapping of active sites (MNPs) and the combination of other 2D materials (nitrides and chalcogenides) with graphene, respectively, to prevent the degradation, corrosion, and dissolution of active sites during electrochemical processes. In addition to HER, these graphene-based materials have high potential for use in various industrial applications such as CO_2_ reduction, energy storage, water purification, electronics, etc. The molecular functionalization of graphene materials, generating various functional groups, can induce certain chemical, physical, and electronic properties by altering the delocalization of the carbon lattice. Moreover, it can prevent the agglomeration of nanosheets and provide a comparatively high surface area, thus facilitating facile electron transfer and access to the increased number of active sites, endowing fast HER kinetics. Importantly, graphene-based electrocatalysts need to be synthesized in bulk to meet the requirements of commercial applications. However, it is difficult to control precisely the uniform distribution of active sites, functionalization, composition, bundling, and morphology. Novel guest functional groups need to be picked, synthesized/modified, and covalently functionalized through various reactions, such as diazotization, cycloaddition, cyanation, silanization, etc., to investigate and subsequently prevent the associated loss in overall activity during the electrolysis process. Importantly, the involvement of harmful chemicals and solvents during the covalent molecular functionalization process needs to be avoided by developing greener processes utilizing less harmful substrates. For instance, the Diels–Alder reaction is generally used to attach molecules with suitable diene groups onto the surface of graphene; however, this process requires harmful chemicals. Interestingly, the same reaction can also be performed using deep eutectic solvents without the involvement of any harmful chemicals or solvents. Hence, the discovery of environmentally friendly methods for the development of rationally engineered electrocatalysts, excluding the requirement of harmful solvents and chemicals, can have a promising future. Moreover, the identification of real active sites in the case of multifunctional electrocatalysts is crucial for a conclusive understanding of substrate absorption and desorption phenomena. Advances in computational chemistry can certainly be helpful to design future electrocatalysts and understand the real-time HER mechanism. It can reveal the nature of the relationship among active sites and substrates and can establish the activity–functionality relationship. Sometimes, active sites can undergo structural changes due to adverse electrochemical reaction conditions. In this case, *in situ* analytical techniques can be beneficial to investigate the reconfiguration of active sites during the HER. Based on the data presented in this review article, future research should focus on improving the stability and lowering the cost of electrocatalysts by excluding noble metals without compromising performance. Other nanomaterials, such as nitrides and phosphides, can be utilized to construct heterostructures with higher stability. Moreover, generating intentional defects by introducing novel active sites, such as single atoms, can effectively participate to tune the electronic properties as well as the stability of the electrocatalyst and enhance its activity. In addition to material research, challenges associated with production cost, scalability, and process sustainability also need to be addressed. Lastly, technological development still requires tremendous effort in the covalent molecular functionalization of graphene-based materials to overcome the associated drawbacks. Certainly, this area has enormous scope and could be explored in the future.

## Acronyms

HER: hydrogen evolution reaction
OER: oxygen evolution reaction
GO: graphene oxide
rGO: reduced graphene oxide
GQDs: graphene quantum dots
MOF: metal–organic framework
COF: covalent–organic framework
FGr: fluorographene
CVD: chemical vapor deposition
MNP: metal nanoparticles
ECSA: electrochemical surface area
COP: covalent organic polymers
TPP: tetrakis­(4-aminophenyl)­porphyrin
DMF: dimethylformamide
TPPCOP: polymeric porphyrins
DCC: dicyclohexylcarbodiimide
HOPG: highly oriented pyrolytic graphite
GA: graphene aerogels
TFB: 1,3,5-benzenetricarboxaldehyde
Bpy: 5,5′-diamino-2,2′-bipyridine
EDA: ethylene diamine
GCE: glassy carbon electrode
CV: cyclic voltammetry
TOFs: turnover frequency
PVP: poly­(vinylpyrrolidone)
NCs: nanocrystals
GNS: graphene nanosheets
HT-AFNG: highly torn amine-functionalized nitrogen-doped graphene
DFT: density functional theory
PPD: *p*-phenylenediamine
PDDA: poly­(diallyldimethylammonium chloride)
ALQD: atom-layer 2H-MoS_2_ nanosheets
ALMS: atom-layered MoS_2_ nanosheets
CoP: cobalt phosphide nanoparticles
S-TiO_2_: nanostepped TiO_2_
ZnTAPc: zinc tetraaminophthalocyanine
SCE: saturated calomel electrode
